# A Simple and Efficient Method for the Partial Synthesis of Pure (3*R*,3’*S*)-Astaxanthin from (3*R*,3’*R*,6’*R*)-Lutein and Lutein Esters via (3*R*,3’*S*)-Zeaxanthin and Theoretical Study of Their Formation Mechanisms

**DOI:** 10.3390/molecules24071386

**Published:** 2019-04-09

**Authors:** Eloy Rodríguez-deLeón, J. Oscar. C. Jiménez-Halla, José E. Báez, M. Moustapha Bah

**Affiliations:** 1 Posgrado en Ciencias Químico Biológicas, Faculty of Chemistry, Autonomous University of Querétaro, Querétaro 76010, Mexico; eloy.q22@gmail.com; 2 Department of Chemistry, Division of Natural and Exact Sciences, University of Guanajuato, Guanajuato 36050, Mexico; jebaez14@yahoo.com.mx

**Keywords:** carotenoids, *meso*-zeaxanthin, (3*R*,3’*S*)-astaxanthin, partial synthesis, DFT calculation, reaction mechanism

## Abstract

Carotenoids are natural compounds that have important roles in promoting and maintaining human health. Synthetic astaxanthin is a highly requested product by the aquaculture industry, but natural astaxanthin is not. Various strategies have been developed to synthesize this carotenoid. Nonetheless, these approaches have not only provided limited global yields, but its main commercial source also carries several health risks for humans. In this contribution, the one-pot base-catalyzed reaction of (3*R*,3’*R*,6’*R*)-lutein (**1**) esters has resulted in a successful isomerization process to easily obtain up to 95% meso-zeaxanthin (**2**), which in turn is oxidized to (3*R*,3’*S*)-astaxanthin (**3**) with a global yield of 68%. The same oxidation performed with UV irradiation (365 nm) for 5 min provided the highest global yield (76%). These chemical transformations have also been achieved with a significant reduction of the health risks associated with its potential human consumption. Furthermore, this is the first time only one of the configurational isomers has been obtained semisynthetically. The poorly understood formation mechanisms of these two compounds were also investigated using Density-Functional Theory (DFT) calculations. These theoretical studies revealed that the isomerization involves a base-catalyzed deprotonation at C-6’, followed by C-4’ protonation, while the oxidation occurs via free radical mechanisms.

## 1. Introduction

**Figure 1:**
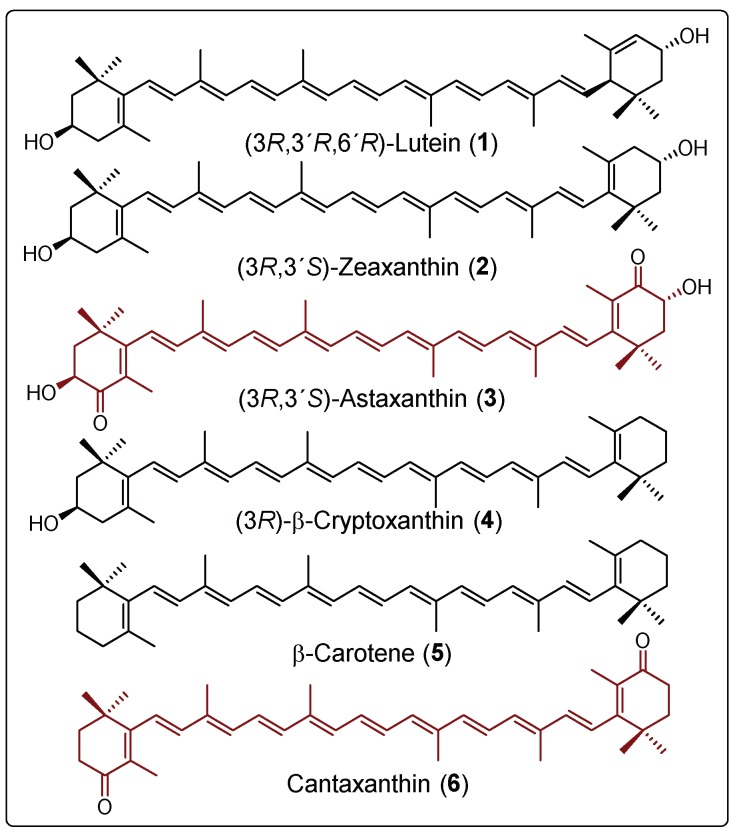
Structures of some common carotenoids.

Carotenoids are natural compounds synthesized by plants and some other photosynthetic organisms such as algae, some types of bacteria, and a small number of aquatic organisms, whereby they have diverse and crucial functions [1]. More than 750 natural carotenoids have been identified in nature [2]. Lutein (**1**), (3*R*,3’*R*)-zeaxanthin, (3*R*)-β-cryptoxanthin (**4**), and β-carotene (**5**) (Figure 1) are among the most abundant [3]. 

**Scheme 1:**
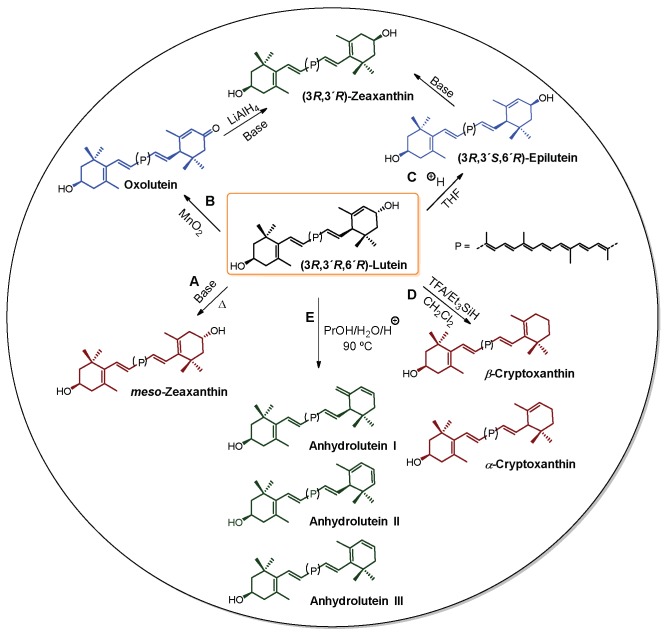
Lutein as the starting material for the partial synthesis of other carotenoids.

These tetraterpenes first gained notability due to their colors. Currently, carotenoids and some apocarotenoids, which are sub-products resulting from the oxidative cleavage of a natural carotenoid, have important commercial applications in industry as nutraceutical additives, vitamin supplements, and cosmetic ingredients, and some have applications in the pharmaceutical industry [4]. For commercial purposes, carotenoids are mainly produced by chemical synthesis or less often by extraction from their natural sources [5,6], as is the case for astaxanthin (**3**), which is extracted from the microalga *Haematoccocus pluvialis*. Commercial synthetic astaxanthin is actually a mixture of the two enantiomers (3*R*,3’*R* and 3*S*,3’*S*) and the meso compound (3*R*,3’*S*) in the ratio of 1:1:2, respectively [7,8]. However, astaxanthin obtained from *H. pluvialis* is mainly obtained in its mono and diesterified forms, and has been reported to account for up to 100% of (3*S*,3’*S*) [8]. Various strategies starting from natural carotenoids have been developed for obtaining other carotenoids or their derivatives, as illustrated in Scheme 1 for the partial synthesis of α-cryptoxanthins and β-cryptoxanthins, anhydroluteins, and zeaxanthins, using compound **1** as the starting material. For example, in the presence of a base and under heating, compound **1** can isomerize to meso-zeaxanthin (Scheme 1, route A) [9,10]. The mechanism by which this chemical transformation occurs has not yet been unambiguously elucidated, since there is only one proposal, which was not sufficiently supported [11]. On the other hand, the oxidation of the allylic hydroxyl group in **1** using MnO_2_ has led to oxolutein (route B) [12], which is further reduced and then isomerized to the diastereoisomeric mixture composed of **2** and (3*R*,3’*R*)-zeaxanthin. Another strategy for obtaining (3*R*,3’*R*)-zeaxanthin has been the epimerization of **1** to 3’-epilutein in acid media [13]. This epimer in turn undergoes isomerization to (3*R*,3’*R*)-zeaxanthin (route C) [14]. This epimerization process has been reported to experimentally occur in green vegetables when processing in an acid medium [15]. Also, Khachik et al. have used **1** as the starting material for the partial synthesis of α-cryptoxanthins and β-crypthoxanthins. They described an industrially viable process to transform **1** into the optically active compound **4** (Figure 1) with a high yield via allylic deoxygenation in which a strong acid (Trifluoroacetic acid: TFA) and different hydride ion donors are employed (route D) [16]. Finally, the high-temperature and acid-catalyzed dehydration of **1** provided a mixture of anhydroluteins (route E) with an overall yield of 86% [17].

Also, carotenoids have gained attention for their beneficial effects on human health [18,19,20]. For example, (3*S*,3’*S*)-astaxanthin is considered one of the most powerful antioxidants in nature [21], and has been reported to possess potential protective attributes against carcinogenesis [22,23], as well as have anti-inflammatory [24], antidiabetic [25,26], and antihypertensive effects [27]. Synthetic astaxanthin and some derivatives with the same skeleton as the central nucleus have been shown to contribute important benefits to the cardiovascular system [28]. An example of this was Cardax, which is the formerly commercialized disodium disuccinate salt of synthetic astaxanthin, and had a positive effect on high blood pressure and a cardioprotective effect [28,29,30]. There are several synthetic as well as extraction methods for obtaining astaxanthin; however, the extraction makes use of the highly expensive supercritical fluid extraction and provides less than 1% [21] of the increasing global demand. In addition to this, its total synthesis has always been achieved after long processes [7], even at the industrial level [1], whereby 52% of the mixture of the three configurational isomers has been reported as the overall better yield after seven synthetic steps [31]. Additionally, only natural astaxanthin is approved for medicinal purposes, owing to safety concerns associated with the potential toxicity of residual reagents, restricting the use of the synthetic compound in aquaculture, where it is employed to provide the pink-red tone to many edible organisms such as salmon, trout, and shrimp [21]. Until now, only one study concerning the allylic oxidation as a strategy for the partial synthesis of astaxanthin has been reported, where zeaxanthin and halogenated reagents were used as starting materials to subsequently oxidize the halogenated derivative [32], although the associated reaction mechanisms and the stereochemistry of the product were not determined. Another proposal is the one-step formation of astaxanthin from cantaxanthin (**6**) [33]. The allylic oxidation process usually involves the use of transition metals, which are not only environmentally unfriendly processes [34], but also carry important risks for human health when consumed [21]. In contrast to the limitations of these methodologies, our contribution has achieved a substantially simple, and more efficient, partial synthesis of zeaxanthin with good yields, employing the less toxic *n*-butanol, whilst avoiding the use of high-pressure and transition metals for obtaining (3*R*,3’*S*)-astaxanthin (**3**) through a radical process. Although the partial synthesis of zeaxanthin and astaxanthin have been described in different patents, to date there has not been any report on the overall yield of this compound starting from lutein or lutein esters. This opens an alternative way of obtaining this highly demanded product and the possibility of investigating its pharmacological benefits as an independent isomer, in contrast to any forms of the commercial astaxanthin products. Furthermore, the mechanisms involved in the isomerization of **1** into **2**, and the oxidation of **2** to **3** were computationally explored in this contribution via DFT calculations.

## 2. Results and Discussion 

### 2.1. Synthesis and Formation Mechanism of Meso-zeaxanthin

#### 2.1.1. High Yield Preparation of *Meso*-zeaxanthin

For the partial synthesis of **2**, the natural lutein esters were first extracted from the oleoresin of marigold (*Tagetes erecta* L.) and then hydrolyzed using an ethanolic KOH solution to provide compound **1**. For the conversion of **1** into **2**, different reaction conditions employing different bases (10 equivalents in each reaction) were tested (Table 1). It was found that the reaction of **1** does not occur at all with bulky bases such as TEA (triethylamine), DBU (1,8-diazabicyclo[5.4.0]undec-7-ene), and DIPEA (*N*,*N*-diisopropylethylamine). Meanwhile, when potassium *tert*-butoxide (*t*-BuOK), another bulky reactant, was added, only dehydration was detected, but isomerization products were not. By contrast, small bases such as potassium and sodium hydroxides (KOH and NaOH) were found to be effective in achieving the isomerization. 

When testing solvents such as methanol, ethanol, or propylene glycol (PG) and KOH, a moderate yield (60–65%) was obtained. Additionally, the reaction performance was found to be improved when *n*-butanol, KOH, and a high temperature (115 °C) were used (Table S3), whereby 92% yield was achieved. Torres-Cardona et al. asserted in a patent that the conversion reaction occurs in ethanol and that the efficiency of the reaction can be improved up to 20% conversion when applying pressure [9]. In another patent, Bernhard et al. used dimethyl sulfoxide (DMSO) as the solvent and reported a yield between 50–80% [10]. This reaction was found to work well, resulting in 75% yield (Table S3, Entry 2).

All the reactions were monitored using HPLC reverse-phase conditions (Figures S1–S3), which consisted of a C_30_ column and gradients of methanol, methyl *tert*-butyl ether (MTBE), and water (Table S1). Based on the best reaction conditions found (Table 1, Entry 4), the isomerization reaction from lutein esters in a one-pot approach was explored (Table 2), and the KOH concentration was increased from 10 to 12 equivalents. Although this process was revealed to be somewhat slower when compared with that starting from **1**, it provided a more satisfactory yield of 95% (Table 2, Entry 2).

#### 2.1.2. Theoretical Mechanism of the Conversion of (3*R*,3’*R*,6’*R*)-Lutein to *Meso*-zeaxanthin

To get some insight into the role of the base, the p*K*a value of the doubly allylic proton in the 6’-position of the α-ionone ring of lutein was calculated. Different calculations using three different functionals allowed the determination of a p*K*a value for this proton between 4.75–5.02 (Table 3). These results demonstrated that the isomerization process does not depend on the p*K*a of the proton in the 6’-position for the isomerization to occur, because the use of a base such as potassium *tert*-butoxide with a p*K*a similar to potassium hydroxide does not promote the isomerization. Thus, it can be argued that the reaction is only possible with small bases due to steric hindrance. 

**Figure 2:**
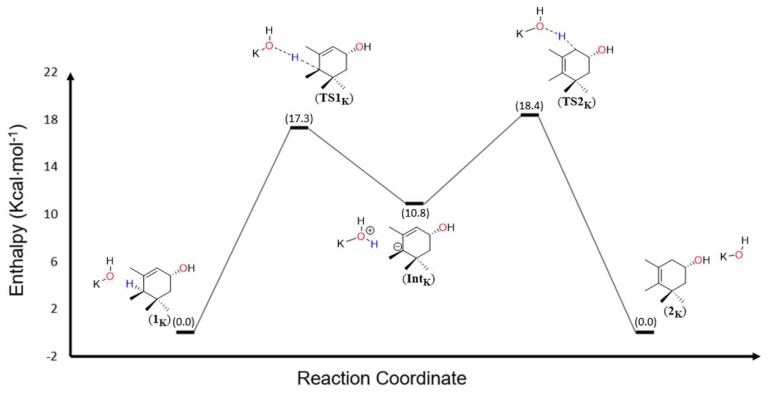
Energy profile calculated at the (PCM:*n*-butanol)M06-L/6-311 + G(2d)//M06-L/6-31G(d) level for the conversion of **1** into **2**.

With regard to the mechanism involved in this isomerization, DFT calculations at the (PCM:*n*-butanol)M06-L/6-311 + G(2d)//M06-L/6-31G(d) level were performed (Figure 2). A model system was considered to conduct the computations, which generated the results reported herein. That is, only one α-ionone ring of **1** was set up. Calculations with the full system (involving the delocalized π system in the chain of double bonds) can be found in the Supporting Information (Figure S15). Since the results using the model system are almost identical to those involving the complete lutein and zeaxanthin molecules, the smaller molecule was investigated for simplicity. The reaction starts as the base (potassium hydroxide) and deprotonates at the 6’-position of the lutein model system (**1_k_**) (Figure 2). This reaction step occurs through the transition state **TS1_k_** with an energy barrier of 17.3 kcal⋅mol^−1^. The reaction occurs with the formation of a carbanion intermediate (**Int_k_**), which is 10.8 kcal⋅mol^−1^ above the energy of the reactants. Then, the intermediate rearranges itself to form a completely conjugated polyene. In the second step, the conjugated base transfers the proton to the allylic carbon through the transition state **TS2_k_**, with an energy barrier of 7.6 kcal⋅mol^−1^. This protonation in the 4’-position leads to the model system of zeaxanthin (**2_k_**), which is an exothermic step (−18.4 kcal⋅mol^−1^). The reaction mechanism using bulkier bases such as DBU and TEA was also computationally explored, from which it could be concluded that more energy is needed for the conversion of **1** into **2**. These theoretical calculations can explain, at least in part, why the experimental assays did not provide any yields, and most probably that the steric hindrance could have prevented the reactions from occuring.

### 2.2. Synthesis and Formation Mechanism of Astaxanthin from Zeaxanthin

#### 2.2.1. Synthesis of (3*R*,3’*S*)-Astaxanthin

For the partial synthesis of **3**, highly purified **2** was used as the starting material, and the allylic oxidation assays were performed employing a catalytic concentration of iodine and hypobromous acid as oxidants (Table 4), in the same way as the reaction conditions proposed by Schloemer et al. [32]. As was observed by these authors, the 3-hydroxyl and 3’-hydroxyl groups’ protection of **2** was not necessary. They used halogenating agents such as *N*-bromo succinamide (NBS) to halogenate the allylic position, which then was oxidized to the carbonyl group by HBrO. In this contribution, we focused only on the use of salts and catalytic quantities of iodine in order to conduct at least a mild eco-friendly process. It is worth mentioning that these salts are easily removed from the reaction medium. These reagents can form free radicals, and therefore probably produce a highly stable allylic radical of compound **2**, which in turn is oxidized in the 4 and 4’ allylic positions. This hypothesis is supported by the theoretical calculations, which indicate that the formation of an allylic radical is preferred over that of a hydroxyl radical at position 3. In these experimental assays, concentrations of iodine as well as the quantities of oxidant salts were varied. Previous investigations indicate that an iodine solution mixed with carotenoids can form radicals, and this has been confirmed by Electron Paramagnetic Resonance (EPR) studies [35,36,37]. Therefore, using different proportions of the oxidant mixture (Table S4), we found that under vigorous stirring in an aqueous solution with two equivalents of sodium bromate and one equivalent of sodium metabisulfite mixed with **2**, the oxidation process occurs with good yields (72%). Likewise, we found that the reaction proceeds in methylene chloride, but not in acetone or water (Table 4). The role of the temperature was also investigated. Room or higher temperatures do not favor the formation of **3**. In contrast, 15 °C or lower temperatures are required for the conversion to occur. However, a very low yield is obtained below 0 °C. These experiments allow an optimal temperature between 0–10 °C for the conversion of **2** to **3** to be determined. The optimal ratio of iodine, which is the second oxidant agent used in the process, was 10% mol. At a major concentration of iodine (20% or more), the reaction still works, but the yield starts to decay. On the other hand, the reaction proceeds at a low concentration of iodine (2 or 5% mol), but the yield is lower than that obtained with 10% mol. In summary, the best conversion was achieved with CH_2_Cl_2_ as the solvent, an optimal temperature of 10 °C, 10% mol of iodine, and 2.5 h of reaction. It is well-known that UV light promotes radical formation. Interestingly, it was found that the reaction occurs without UV light, although the yield was improved by 8% when this radiation (365 nm, common commercial laboratory lamp for 5 min) was applied. It is worth mentioning that in CH_2_Cl_2_ solution, all *trans*-astaxanthin undergoes reversible isomerization to 9-*cis*- and 13-*cis* astaxanthins, so that the commercial product, including that from *H. pluvialis*, is a mixture of three geometric isomers [38,39]. This phenomenon was observed during the HPLC analysis of the final product (**3**) (Figure S4), which was obtained with a global yield of 76% from lutein esters. 

#### 2.2.2. Theoretical Mechanism for the Conversion of (3*R*,3’*S*)-Zeaxanthin to (3*R*,3’*S*)-Astaxanthin

With regard to the mechanism involved in this oxidation, we performed theoretical calculations at the same theoretical level as was used and described above. However, because our proposal involves radical reaction steps as described by Coote et al. [40], we do not provide energy barriers for these transformations, since our level of theory is not accurate enough to discuss their kinetics. Moreover, the radical species indicated here are neutral doublets for which open-shell calculations were performed, whereas the non-radical compounds are neutral singlets for which restricted open-shell calculations were applied for consistency.

**Scheme 2:**
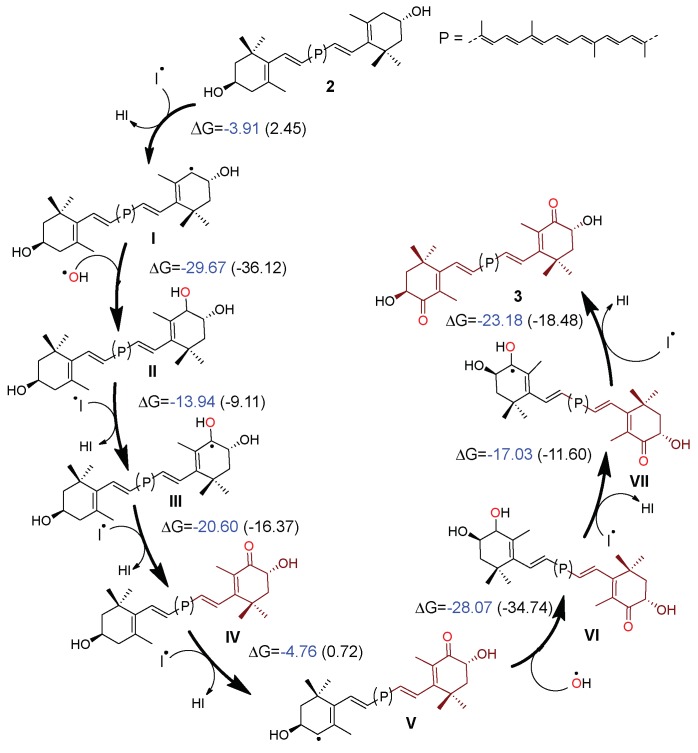
Plausible radical mechanism for the allylic oxidation of **2** to **3**.

We suggest that the reaction is initiated by the formation of the allylic radical **I** at the 4’-position of **2** (Scheme 2), and subsequently that the reaction with the hydroxyl radical to form the di-hydroxylated compound **II** is achieved. 

From here, there are two possible routes to follow: 1) the formation of a new allylic radical at C-4, or 2) the formation of a hydroxyl allylic radical at C-4’ (**III**) (Scheme 2). The formation of **III** (ΔG = −13.94 kcal/mol) is more favored than the other (**III_1_** in pathway 1, Scheme S; (ΔG = −3.54 kcal/mol). The subsequent reaction between the iodine radical and the hydrogen of the hydroxyl group at 4’-position, followed by the formation of a carbonyl group conjugated with the double bond in the positions 5(6) in **IV,** is favored. Later, a new allylic radical (**V**) is formed as a result of the reaction between **IV** and the iodine radical. In the next step, **V** reacts with the hydroxyl radical to form the hydroxyl-ketone **VI**. Finally, the reaction between **VI** and a radical iodine gives rise to the hydroxyl allylic radical **VII** formation, which undergoes the deprotonation promoted by iodine radicals, leading to the final compound **3**.

## 3. Materials and Methods

### 3.1. General Experimental Procedures

^1^H, ^13^C, and two-dimensional NMR spectra were acquired on a Bruker Avance III HD 500-MHz instrument, using CDCl_3_ as solvent and tetramethylsilane (TMS) as internal standard. HPLC analyses were carried out on a Waters apparatus (Waters Chromatography Divison, Milford, MA, USA) composed of a 2695e multi-solvent delivery system and a 2998 Photodiode Array (PDA) detector, using a C_30_ column (5 μm, 250 × 4.5 mm) at a flow rate of 0.7 mL/min. Control of this equipment, data acquisition, processing, and the management of chromatographic information were performed by the Empower software (Waters) (Waters Chromatography Divison, Milford, MA, USA). Thin Layer Chromatography (TLC) analyses were performed on 0.25-mm silica gel PF 254 (Merck) (Merck, Kenilworth, NJ, USA) plates, and spots were visually defined. All the salts, such as potassium hydroxide, sodium hydroxide, potassium *tert*-butoxide, sodium carbonate, potassium carbonate, sodium bromate, sodium metabisulfite, as well as citric acid and iodine, were purchased from REACTIVOS QUIMICA MEYER (Tlahuac, Ciudad de México, Mexico). The organic bases such as TEA, DIPEA, and DBU were purchased from Sigma Aldrich Co. (St. Louis, MO, USA). HPLC and analytical solvents were acquired from Baker-Mallincrodt (JT Baker, Mallinckrodt Baker Inc, Phillipsburg, NJ, USA). The marigold oleoresin was purchased from Oleo-especias (Zapopan, Jalisco, Mexico). For the extraction of lutein esters, the marigold oleoresin (100 g) was washed successively with 50 mL of isopropyl alcohol and 50 mL of hexane. Each washing process was repeated three times. Then, the final residue was dried at room temperature, giving 65 g of dry starting material enriched in lutein esters.

### 3.2. Free (3R,3’R,6’R)-Lutein (***1***) from Oleoresin

The marigold oleoresin (100 g) was hydrolyzed with KOH (11.2 g) in ethanol (EtOH) (125 mL) under reflux for 3 hours. Upon completion of the reaction, the mixture was neutralized with 10.7% aqueous H_3_PO_4_ (*v*/*v*) and washed with 100 mL of hexane. About 12.5 g of a dry orange-red solid was recovered and purified by precipitation by solvent exchange from dichloromethane to cold methanol. The compound obtained was identified as (3*R*,3’*R*,6’*R*)-lutein (**1**): ^1^H-NMR (500 MHz, CDCl_3_) δ (ppm) (Figure S6): 0.85 (3H, s, H-16’), 1.00 (3H, s, H-17’), 1.07 (6H, s, H16 and H-17), 1.36 (1H, dd, *J* = 13.1, 7.0 Hz, H-2’_A_), 1.48 (1H, t, *J* = 12.0 Hz, H-2_A_), 1.63 (3H, s, H-18’), 1.74 (3H, s, H-18), 1.78 (1H, m, H-2), 1.84 (1H, dd, *J* = 13.1, 7.0 Hz, H-2’_B_), 1.91 (3H, s, H-19’), 1.97 (9H, s, H-19, H-20, and H-20’), 2.05 (1H, dd, *J* = 16.5, 9.5 Hz, H-4_A_), 2.39 (1H, m, H-4_B_), 2.41 (1H, d, *J* = 8.1 Hz, H-6’), 4.01 (1H, m, H-3), 4.25 (1H, s, H-3’), 5.44 (1H, dd, *J* = 15.5, 10.0 Hz, H-7’), 5.55 (1H, s, H-4’), 6.12 (3H, m, H-7, H-8, and H-8’), 6.15 (2H, m, H-10 and H-10’), 6.24 (1H, m, H-14), 6.26 (1H, m, H-14’), 6.35 (1H, m, H-12), 6.37 (1H, m, H-12’), 6.64 (4H, m, H-11, H-11’, H-15, and H-15’). ^13^C-NMR (125 MHz, CDCl_3_) δ (ppm) (Figure S7): 12.8 (C-20 and C-20’), 13.1 (C-19 and C-19’), 21.4 (C-18), 22.9 (C-18’), 24.3 (C-17’), 28.7 (C-17), 29.5 (C-16’), 30.3 (C-16), 34.0 (C-1’), 37.1 (C-1), 42.6 (C-4), 44.7 (C-2), 48.4 (C-2’), 54.9 (C-6’), 65.1 (C-3), 65.9 (C-3’), 124.5 (C-4’), 124.8 (C-11’), 124.9 (C-11), 125.6 (C-7), 126.2 (C-5), 128.7 (C-7’), 130.0 (C-15), 130.1 (C-15’), 130.8 (C-10), 131.3 (C-10’), 132.6 (C-14 and C-14’), 135.1 (C-9), 135.7 (C-9’), 136.4 (C-13’), 136.5 (C-13), 137.6 (C-12), 137.7 (C-12’), 137. 8 (C-5’), 138.0 (C-6), 138.5 (C-8 and C-8’). These data match those published by Otaka et al. [41].

### 3.3. Synthesis of (3R,3’S)-Zeaxanthin (***2***)

To 1 mmol (569 mg) of **1** in 10 ml of *n*-butanol, 561 mg of KOH was added, and the solution was heated for 12 h at 115 °C with vigorous stirring. The reaction was monitored by HPLC. Once 90% of the conversion was reached, the reaction was stopped by adding 2N of H_3_PO_4_ until it resulted in a neutral pH. A slight excess of water was added; then, the reaction mixture was filtered, and solvent was removed by rotary evaporation until dry to give a crude residue (540 mg). The compound was recrystallized using methanol, affording orange crystals (yield 92%). This compound was identified as (3*R*,3’*S*)-zeaxanthin (**2**): ^1^H-NMR (500 MHz, CDCl_3_) δ (ppm) (Figure S8): 1.07 (12H, s, H-16, H-16’, H-17, and H-17’), 1.48 (2H, t, *J* = 12.0 Hz, H-2_A_), 1.74 (6H, s, H-18 and H-18’), 1.77 (2H, m, H-2_B_), 1.97 (12H, s, H-19, H-19’, H-20, and H-20’), 2.04 (2H, dd, *J* = 16.5, 5.0 Hz, H-4_A_), 2.39 (2H, dd, *J* = 16.5, 5.0 Hz, H-4_B_), 4.00 (2H, m, H-3 and H-3’), 6.12 (2H, m, H-7 and H-7’), 6.15 (2H, m, H-8 and H-8’), 6.17 (2H, m, H-10 and H-10’), 6.26 (2H, m, H-14 and H-14’), 6.37 (2H, d, *J* = 14.5 Hz, H-12 and H-12’), 6.62 (2H, m, H-15 and H-15’), 6.64 (2H, m, H-11 and H-11’). ^13^C-NMR (125 MHz, CDCl_3_) δ (ppm) (Figure S9): 12.8 (C-20 and C-20’), (C-19 and C-19’), 21.6 (C-18 and C-18’), 28.7 (C-16 and C-16’), 30.3 (C-17 and C-17’), 37.1 (C-1 and C-1’), 42.6 (C-4 and C-4’), 48.4 (C-2 and C-2’), 65.1 (C-3 and C-3’), 124.9 (C-11 and C-11’), 125.6 (C-7 and C-7’), 126.2 (C-5 and C-5’), 130.1 (C-15 and C-15’), 131.3 (C-10 and C-10’), 132.6 (C-14 and C-14’), 135.7 (C-9 and C-9’), 136.4 (C-13 and C-13’), 137.6 (C-12 and C-12’), 137. 8 (C-6 and C-6’), 138.6 (C-8 and C-8’) [42].

### 3.4. Synthesis of (3R,3’S)-astaxanthin (***3***)

Compound **2** (285 mg) was dissolved in methylene chloride (15 mL) and 2.5 mg of iodine was added. The mixture was stirred for 30 min. The oxidant solution was independently prepared according to the following procedure: 100 mg of NaBrO_3_ were dissolved in 5 mL of water and 50 mg of NaHCO_3_ was added; independently, 56 mg of Na_2_S_2_O_5_ was dissolved in 2 mL of water. Both solutions were cooled to 5 °C and mixed. The solution was acidified with citric acid. After 30 s, the solution turned pale yellow, and at this point, it was added to the zeaxanthin-iodine solution. The reaction mixture, which was vigorously stirred and monitored by TLC, over time turned dark red. Once no change in this color was observed, the reaction was stopped, left to reach room temperature, and then neutralized with a 25-mL aqueous solution containing 250 mg of NaHCO_3_ and 80 mg of Na_2_S_2_O_5_. The mixture was extracted with dichloromethane (3 × 15 mL), dried over anhydrous Na_2_SO_4_, filtered, and concentrated under reduced pressure. The residue was purified by preparative thin-layer chromatography on silica gel plates eluted with hexane-acetone (8:2), to afford a 72% yield of a dark red powder. This compound was identified as (3*R*,3’*S*)-astaxanthin (**3**). ^1^H-NMR (500 MHz, CDCl_3_) δ (ppm) (Figure S10): 1.21 (6H, s, H-16 and H-16’), 1.32 (6H, s, H-17 and H-17’), 1.81 (2H, m, H-2_A_ and H-2’_A_), 1.94 (6H, s, H-18 and H-18’), 1.99 (6H, m, H-20 and H-20’), 2.00 (6H, m, H-19 and H-19’), 2.17 (2H, m, H-2_B_ and H-2’_B_), 4.33 (2H, dd, *J* = 14.0, 5.5 Hz, H-3 and H-3’), 6.20 (2H, m, H-7 and H-7’), 6.29 (2H, m, H-14 and H-14’), 6.31 (2H, m, H-10 and H-10’), 6.43 (2H, m, H-8 and H-8’), 6.45 (2H, d, *J* = 7.5 Hz, H-12 and H-12’), 6.66 (2H, m, H11 and H-11’), 6.68 (2H, m, H-15 and H-15’). ^13^C-NMR (125 MHz, CDCl_3_) δ (ppm) (Figure S11): 12.6 (C-19 and C-19’), 12.8 (C-20 and C-20’), 14.0 (C-18 and C-18’), 26.2 (C-17 and C-17’), 30.7 (C-16 and C-16’), 36.8 (C-1 and C-1’), 45.4 (C-2 and C-2’), 69.2 (C-3 and C-3’), 123.3 (C-7 and C-7’), 124.6 (C-11 and C-11’), 126.8 (C-5 and C-5’), 130.7 (C-15 and C-15’), 133.8 (C-14 and C-14’), 134.8 (C-9 and C-9’), 135.2 (C-10 and C-10’), 136.7 (C-13 and C-13’), 139.7 (C-12 and C-12’), 142.4 (C-8 and C-8’), 162.3 (C-6 and C-6’), 200.4 (C-4 and C-4’) [43]. 

### 3.5. Computational Calculations

Gas-phase DFT calculations using the Gaussian09 series (Gaussian Inc., Wallingford, CT, USA) programs were performed [44]. Geometry optimizations were conducted without symmetry restrictions, but using a model system. That is, instead of the full lutein molecule, only one terminal ring was considered in the case of the isomerization reaction. However, the energy of the entire lutein molecule was also calculated for comparison with our model system (Figures S15 and S16). For numerical precision, the local hybrid density-functional M06−L [45] was set up along with a double-ζ quality basis set, 6-31G(d). Also, harmonic frequency calculations were performed at the same level of theory in order to fulfill two objectives. (i) The first was to characterize the nature of the critical points found on the potential energy surface—that is, local minima that contain zero negative (imaginary) Hessian eigenvalues, whereas maxima (transition states) present one and only one negative eigenvalue, which corresponds to the desired reaction coordinate. (ii) The second objective was to obtain the thermal and entropic corrections, which were set up to 298 K and 1 atm. Single-point calculations were later performed over the optimized geometries using the polarizable continuum model (PCM), [46,47,48] defining *n*-butanol as the reaction solvent (ε = 17.332) to obtain the solvation correction at the same level of theory as for the geometric optimizations. In addition, to improve the final energy values reported in this work, we carried out single-point calculations with a triple-ζ quality basis set, 6-311 + G(2d), over each optimized geometry. Thus, the selected level of theory is defined as PCM:*n*-butanol)M06−L/6-311 + G(2d)//M06−L/6-31G(d). For the mechanism involved in the oxidation reaction, we reported our calculations at the same level of theory, but only in the gas phase.

Regarding the p*K*a calculations, we performed geometry optimizations using the PCM model according to Equation (1), where A−H stands for lutein. Thus, the solvated free energy could be calculated as shown in Equation (2), where $G_{solv}\left(H^{+}\right)$ = 264.5 kcal⋅mol^−1^ is the solvation free energy of a proton in *n*-butanol [49] and RTln(24.46) = 1.89 kcal⋅mol^−1^, which is the factor that accounts for the change in the gas-phase reference state from 1 atm to 1 M. The term $G_{gas}\left(H^{+}\right)$ is the absolute free energy of the gas-phase proton at standard temperature and pressure. This energy was easily calculated using the Sackur–Tetrode equation, and has a value of −6.28 kcal/mol, rendering it straightforward to calculate gas-phase acidities.

(1) $$A-H\to A^{-}+H^{+}$$

(2) $$\Delta G_{\mathrm{solv}}=G_{\mathrm{solv}}\left(A-H\right)-\;G_{solv}\left(A^{-}\right)\;-\;G_{solv}\left(H^{+}\right)\;-\;G_{gas}\left(H^{+}\right)+\;\mathrm{RT}\cdot \ln(24.46)$$

So, the p*K*a can then be calculated following Equation (3):

(3) $$pKa\;=\;\frac{\Delta G_{solv}}{2.303\;RT}$$

Finally, these p*K*a calculations were performed using three density functionals: M06-L [45], cam-B3LYP [50,51], and ωB97X-D [52], in conjunction with the basis sets, i.e. 6-31 + G(d), 6-311 + G(2d), and def2-svp.

## 4. Conclusions

A facile, efficient, and mild eco-friendly process to obtain (3*R*,3’*S*)-astaxanthin (**3**) from (3*R*,3’*R*,6’*R*)-lutein (**1**) and lutein esters via (3*R*,3’*S*)-zeaxanthin (**2**) has been developed. The experimental and computational studies of the conversion of **1** into **2** indicated that this process is not favored when using bulky bases. Also, it has been proven that the oxidation of **2** to **3** proceeds without the need for protecting groups, peroxides, or metals. The global yield of **3** was 73% from **1** and 76% from lutein esters, which was substantially higher than those reported in a handful of articles on their partial synthesis, and exceeding the highest yield reported on an industrial scale performed by total synthesis. The suitability of astaxanthin obtained via this process for potential human consumption may now be investigated. Finally, DFT calculations, in addition to leading to an improvement of the yield as a result of UV irradiation, support a plausible radical free mechanism for this conversion.

## Figures and Tables

**Table 1:**
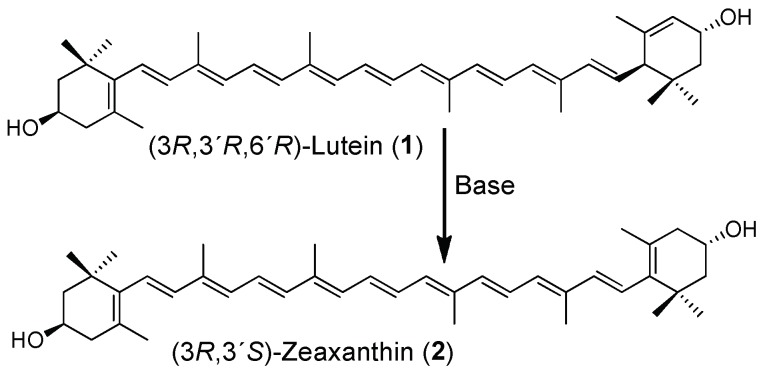
Optimization of the reaction conditions.

Entry	Base	Solvent	Temperature	Time (Hours)	Yield (%)
**1**	KOH	MeOH	Reflux	12	65
**2**	KOH	EtOH	Reflux	12	60
**3**	KOH	Water	Reflux	16	nr
**4**	KOH	*n*-BuOH	115	12	92
**5**	KOH	PG	115	12	62
**6**	NaOH	*n*-BuOH	115	12	85
**7**	DBU	*n*-BuOH	100	20	nr
**8**	TEA	*n*-BuOH	100	24	nr
**9**	^a^ K_2_CO_3_	*n*-BuOH	115	12	22
**10**	DIPEA	*n*-BuOH	115	24	nr
**11**	*t*-BuOK	*n*-BuOH	115	20	- ^b^

nr = no reaction, ^a^ K_2_CO_3_: potassium carbonate, ^b^ dehydration products (anhydroluteins).

**Table 2:**
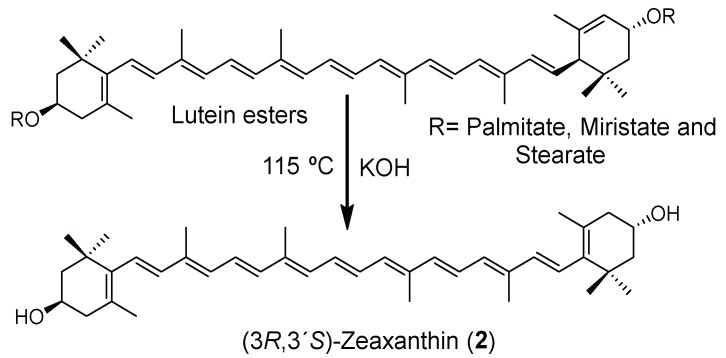
One-pot reaction conditions starting from lutein esters to meso-zeaxanthin.

Entry	Base	Solvent	Time(Hours)	Yield (%)
**1**	KOH	*n*-BuOH	12	86
**2**	KOH	*n*-BuOH	16	95
**3**	KOH	PG	16	72

**Table 3:**
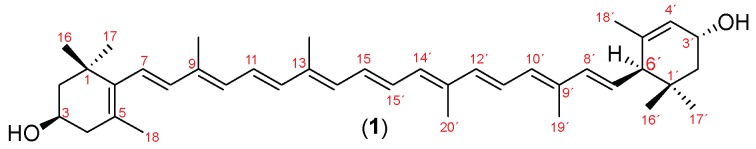
p*K*a values of H-6’ in (3*R*,3’*R*, 6’*R*)-lutein (**1**) calculated with different functionals and basis sets.

	DFT Method	6-311 + G(2d)	6-31 + G(d)	Def2-svp
**1**	M06L	4.75	4.92	4.80
**2**	cam-b3lyp	4.87	4.90	4.90
**3**	ωB97XD	4.89	5.02	4.94

**Table 4:**
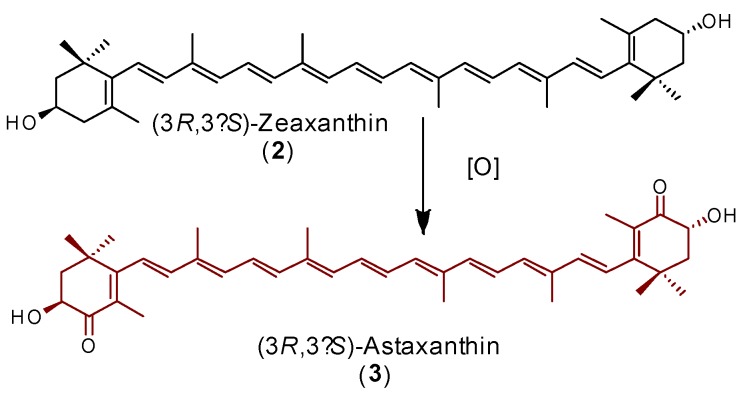
Oxidation reaction of (3*R*,3’*S*)-zeaxanthin to (3*R*,3’*S*)-astaxanthin.

Entry	I_2_ % mol	Solvent	Time Hours	Yield (%)
**1**	20	CH_2_Cl_2_	4	46
**2**	10	CH_2_Cl_2_	4	52
**3**	10	CH_2_Cl_2_	2.5	72
**4**	5	CH_2_Cl_2_	2.5	55
**5**	2	CH_2_Cl_2_	2.5	60
**6**	10	CH_2_Cl_2_	2.5	80 ^a^
**7**	10	acetone	6	nr ^b^
**8**	10	water	6	nr ^b^

^a^ Reaction applying UV light irradiation; ^b^ nr: no reaction.

## Supplementary Materials

Supplementary materials are available online at https://www.mdpi.com/1420-3049/24/7/1386/s1.
