# Supplementary material for: A Simple and Efficient Method for the Partial Synthesis of Pure (3R,3’S)-Astaxanthin from (3R,3’R,6’R)-Lutein and Lutein Esters via (3R,3’S)-Zeaxanthin and Theoretical Study of Their Formation Mechanisms
Source: Molecules. 2019 Apr 9;24(7):1386. doi: 10.3390/molecules24071386 (PMC6480186; doi:10.3390/molecules24071386)
Supplement: Supplementary file 1 [file molecules-24-01386-s001.pdf]

Supplementary materials

A Simple and Efficient Method for the Partial Synthesis of Pure (3*R*,3′*S*)-Astaxanthin from (3*R*,3′*R*,6′*R*)-Lutein and Lutein Esters via (3*R*,3′*S*)-Zeaxanthin and Theoretical Study of their Formation Mechanisms

Eloy Rodríguez-deLeón [1], J. Oscar. C. Jiménez-Halla [2,*], José E. Báez [2] and M. Moustapha Bah [1,*]

[1] Posgrado en Ciencias Químico Biológicas, Faculty of Chemistry, Autonomous University of Querétaro, 76010 Querétaro, Mexico; eloy.q22@gmail.com

[2] Department of Chemistry, Division of Natural and Exact Sciences, University of Guanajuato, 36050 Guanajuato, Mexico; jebaez14@yahoo.com.mx

* Correspondence: jjimenez@ugto.mx (J.O.C.J.-H.); moubah@uaq.mx (M.M.B); Tel.: +52-473-732-0006 (ext. 1433) (J.O.C.J.-H.); +52-442-192-1200 (ext. 5536) (M.M.B)

Contents

HPLC Analysis …………………………………………………………………………… 1

Implementation of the reaction conditions …………………………………………………. 4

^{1}H and ^{13}C NMR spectra…………………………………………………………………... 5

Calculated reaction mechanisms…………………………………………………………… 9

Tables of optimized Cartesian coordinates of the isomerization reaction ……………………... 11

Oxidation reaction mechanism enthalpy and free energy values (Scheme S1) ……………… 22

Tables of optimized Cartesian coordinates of the isomerization reaction ……………………. 23

HPLC Analysis

Table S1. HPLC analytical conditions: Column: C_{30}, 5µm, 140Å, 250 x 4.6 mm; flow rate: 0.7 mL/min; detection at λ = 474 nm; injection volume: 10 µL, run time: 35 minutes; mobile phase: gradient of MeOH–methyl *ter*-butyl ether (MTBE)–H_2O as follow:

Time (minutes)	MeOH (%)	MTBE (%)	H_2O (%)
0	81	15	4
15	66	30	4
23	16	80	4
27	16	80	4
27.1	81	15	4
35	81	15	4

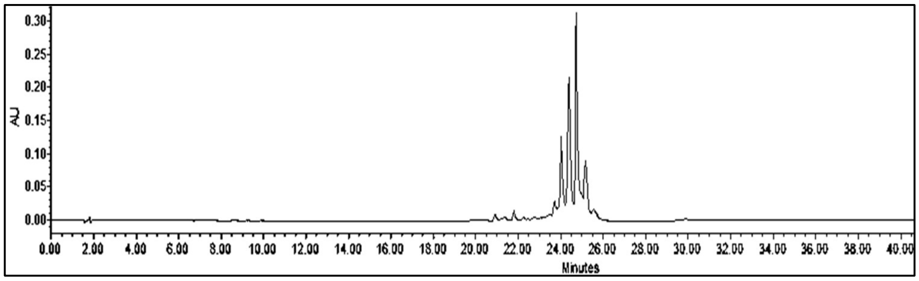

Figure S1. Chromatogram of the lutein esters extracted from the marigold oleoresin.

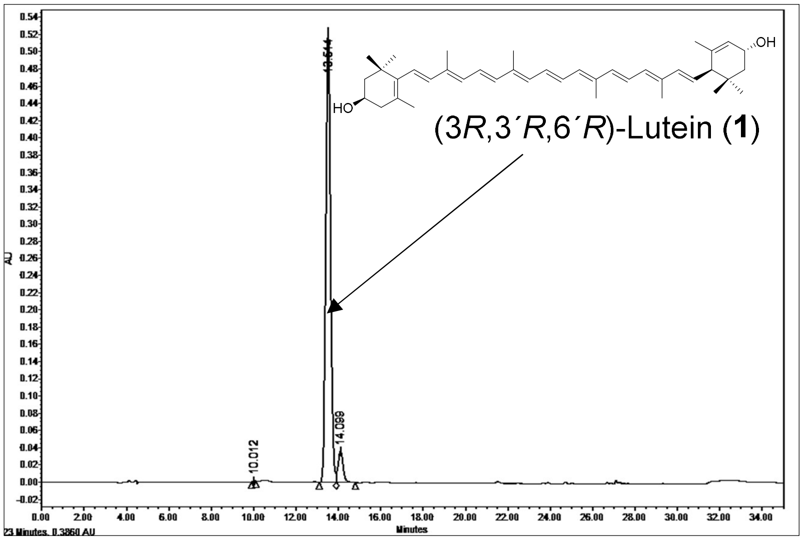

Figure S2. Chromatogram of lutein obtained from KOH-hydrolized lutein esters.

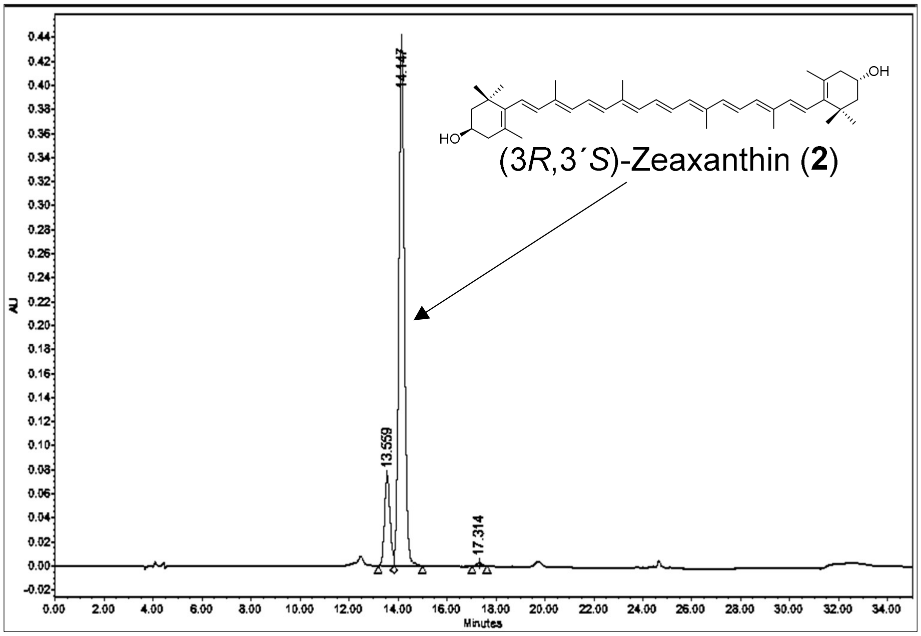

Figure S3. Chromatogram of zeaxanthin obtained from **1**.

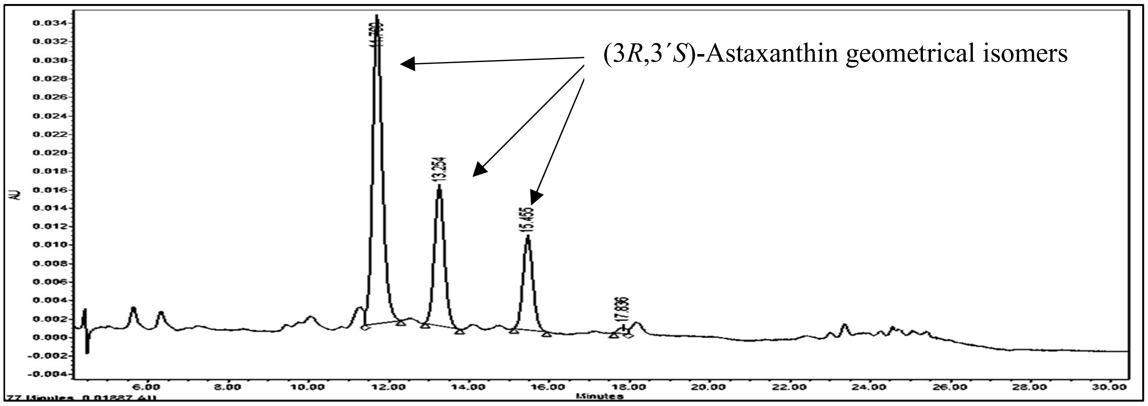

Figure S4. Chromatogram of the (3*R*,3´*S*)-astaxanthin obtained during the partial synthesis. The 3 peaks emerge as a consequence of the inherent *cis*-*trans* isomerization.

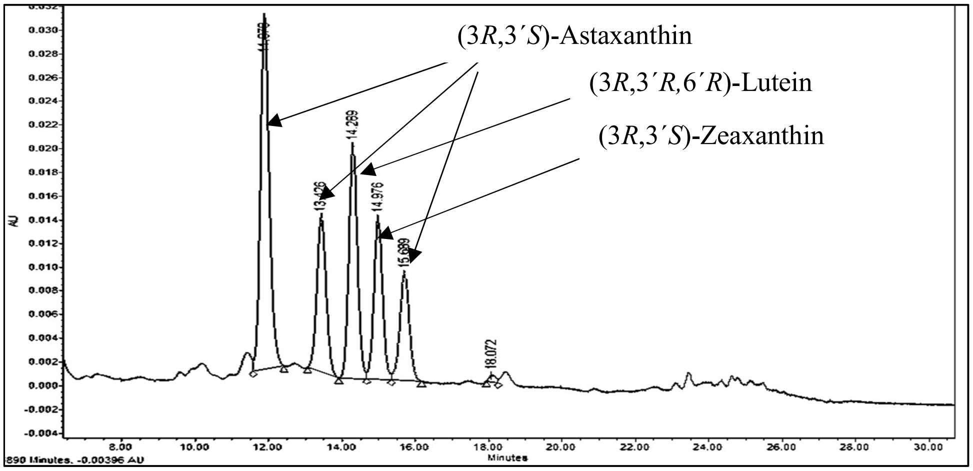

Figure S5. HPLC coelution of the obtained lutein, zeaxanthin and astaxanthin.

Table S2. Experimental UV λ_{max} of the carotenoids.

Compound	λ_{max} (nm)
Lutein	446 & 474
Lutein esters	446 & 474
Zeaxanthin	450 & 478
Astaxanthin	474

Implementation of the reaction conditions

Table S3. Implementation of the reaction conditions for the conversion of **1** to **2**.

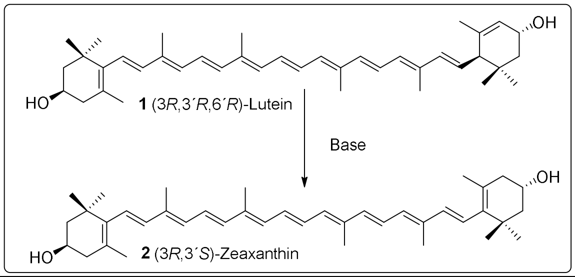

Entry	Reaction time (Hours)	Solvent	Temperature (°C)	KOH (equivalents)	Yield (%)
1	8	No solvent	110	10	30
2	10	DMSO	110	10	75
3	12	Hexanes	Reflux	16	nr
4	14	Toluene	Reflux	12	nr
5	14	*n*-BuOH	115	10	92
6	16	*n*-BuOH	90	10	50
7	24	*n*-BuOH	rt	10	nr
8	12	*n*-BuOH	115	12	92

nr= no reaction

Table S4. Implementation of the reaction conditions for the conversion of **2** to **3**.

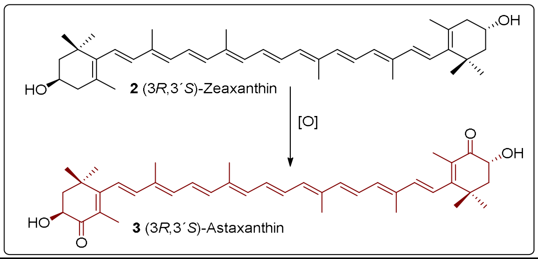

Entry	I_2 % mol	Solvent	Oxidant mixture Equivalents*	Reaction Time (Hours)	Yield (%)
**1**	20	CH_2Cl_2	2:1	4	20
**2**	10	CH_2Cl_2	1.5:1	4	10
**3**	10	CH_2Cl_2	2:1	2	64
**4**	5	CH_2Cl_2	1:1	2.5	5
**5**	2	CH_2Cl_2	4:1	2.5	10
**6**	10	CH_2Cl_2	2:1	4	42
**7**	10	acetone	2:1	6	nr
**8**	10	Water	2:1	6	nr

*Ratio between $NaBrO_3$ with $Na_2S_2O_5$, all reactions at 10 ºC, nr= no reaction.

^{1}H and ^{13}C NMR spectra

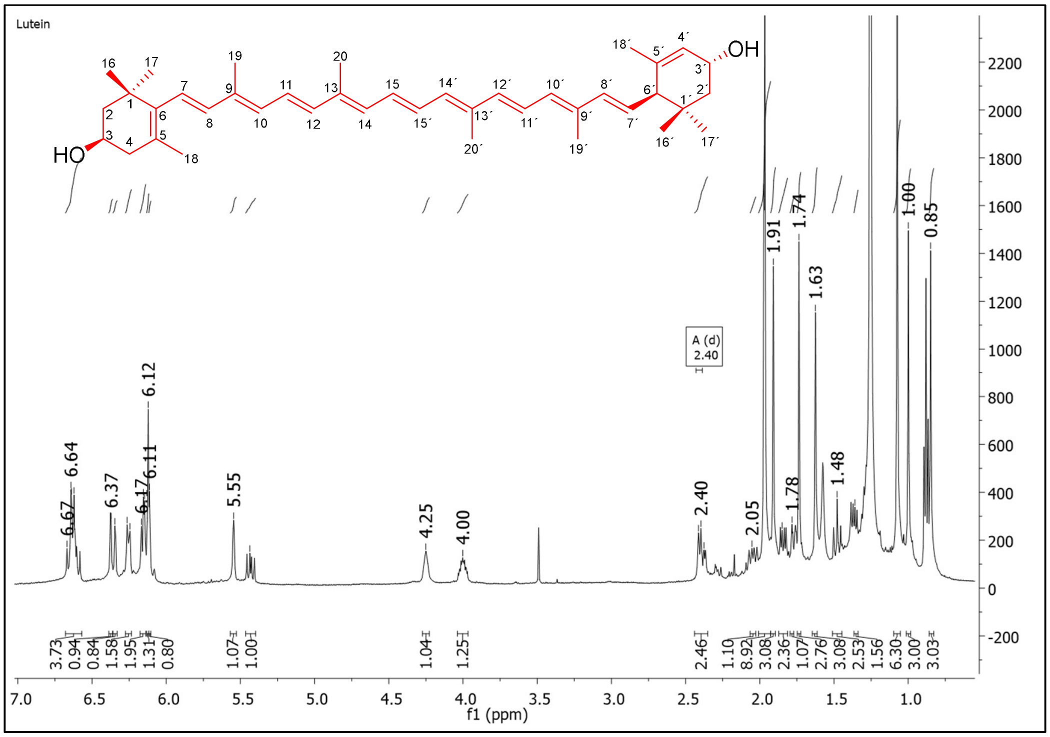

Figure S6. ^{1}H NMR spectrum of (3*R*,3′*R*,6′*R*)-lutein.

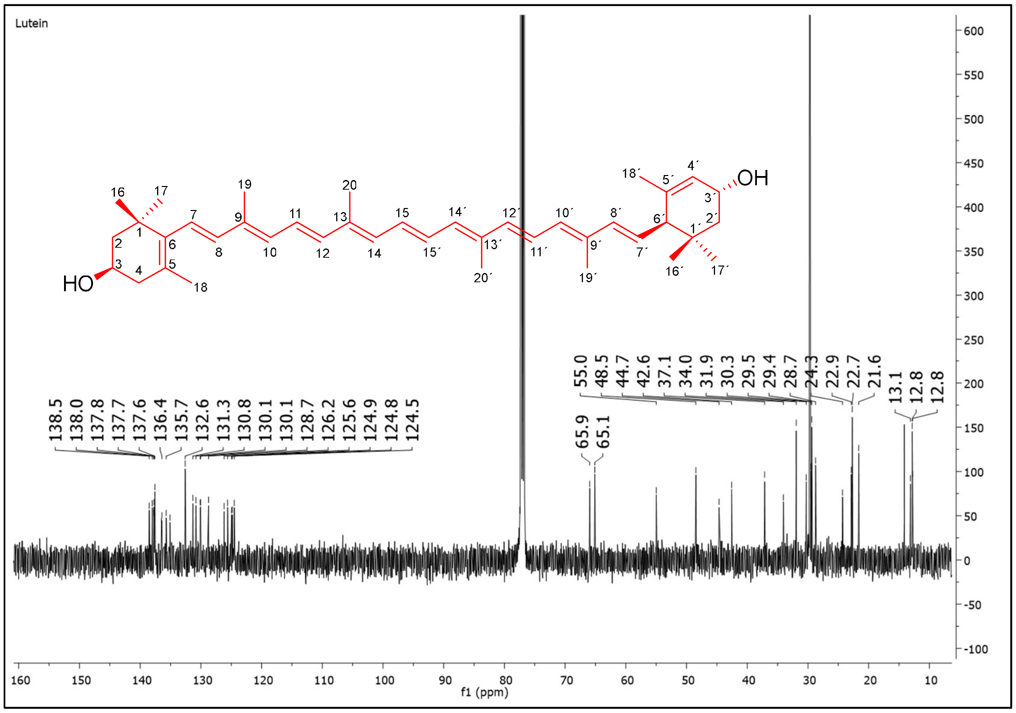

Figure S7. ^{13}C NMR spectrum of (3*R*,3′*R*,6′*R*)-lutein.

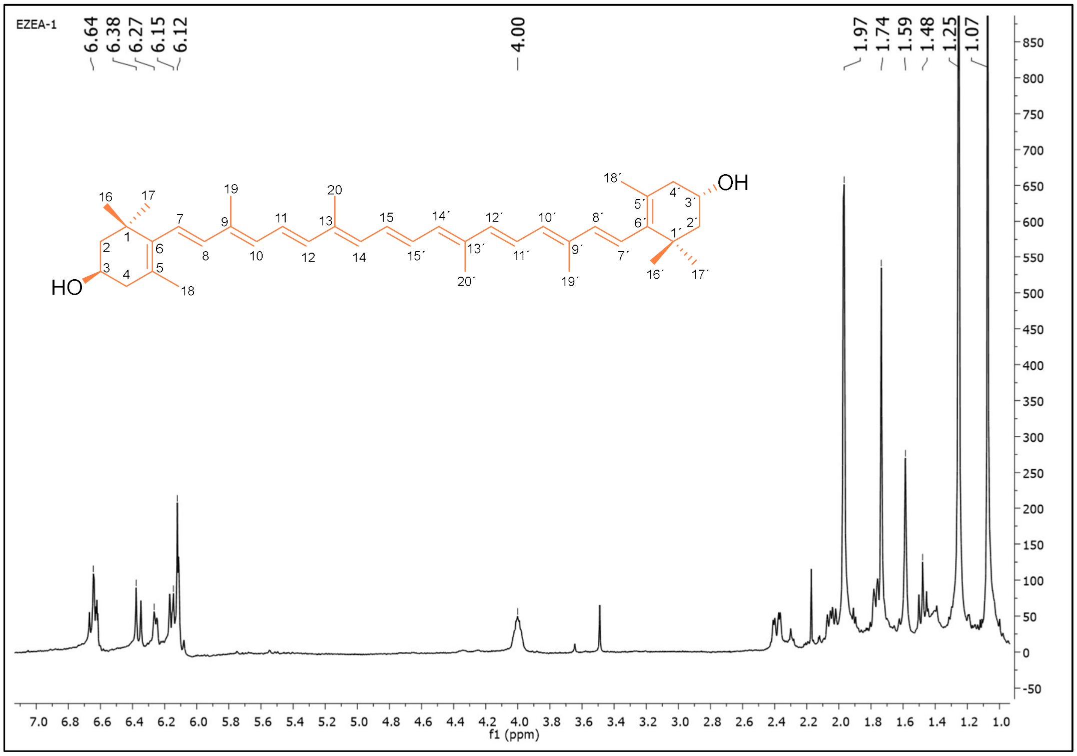

Figure S8. ^{1}H NMR spectrum of of (3*R*,3´*S*)-zeaxanthin.

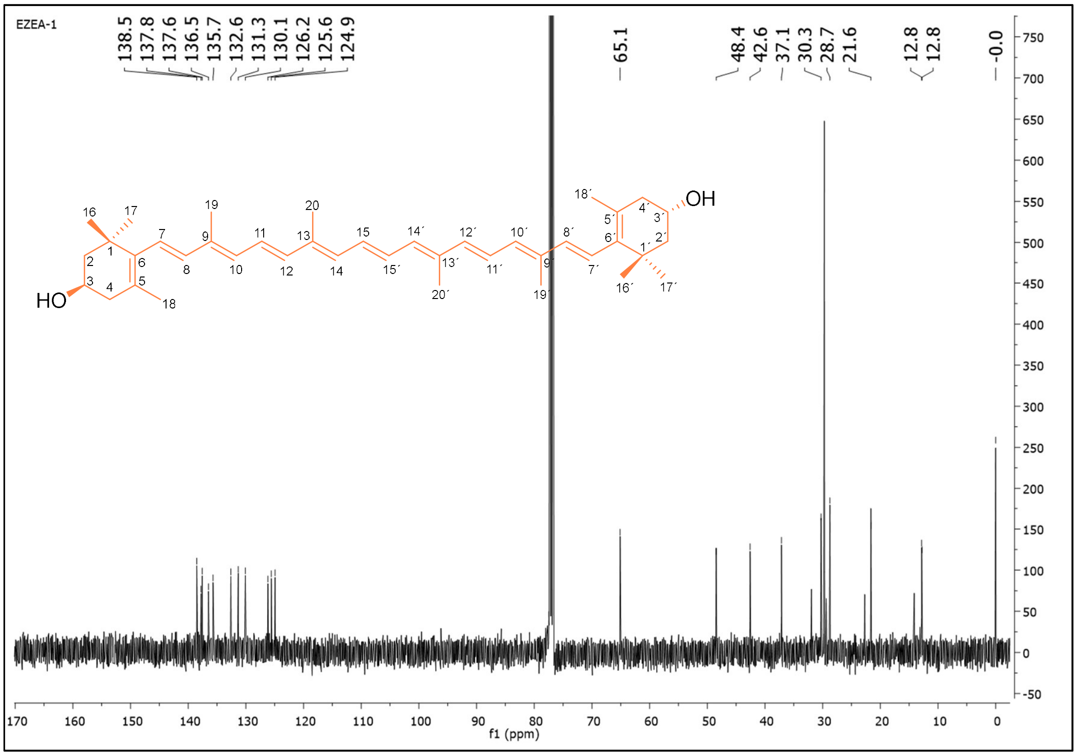

Figure S9. ^{13}C NMR spectrum of (3*R*,3´*S*)-zeaxanthin.

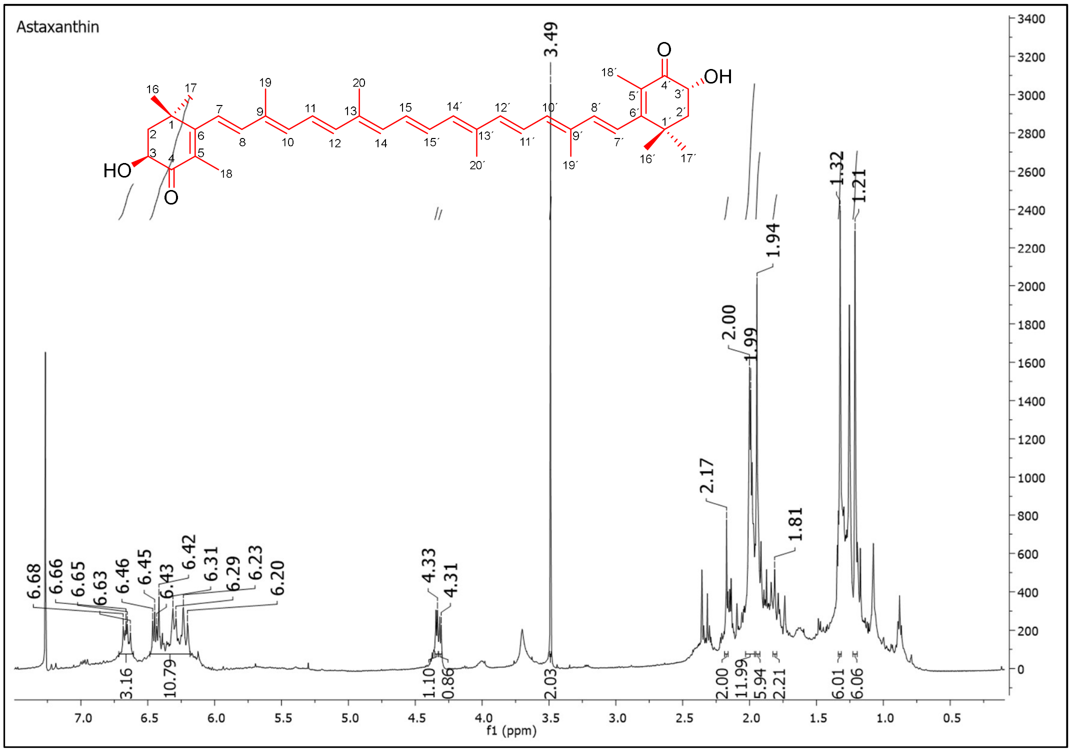

Figure S10. ^{1}H NMR spectrum of (3*R*,3´*S*)-astaxanthin.

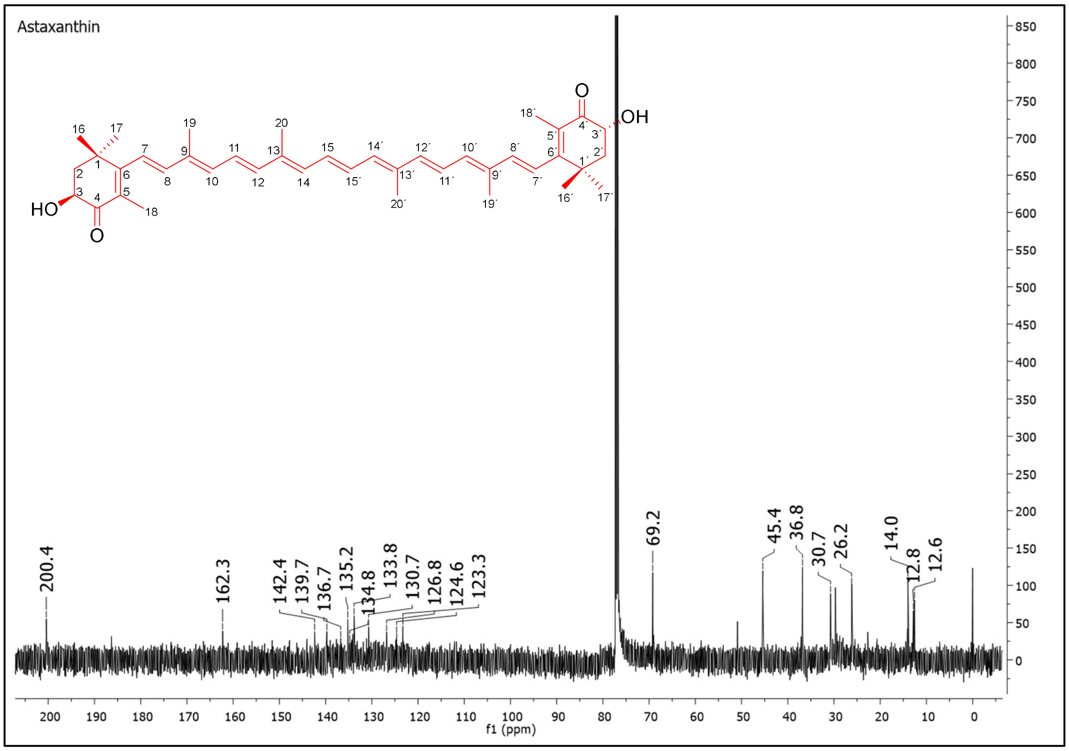

Figure S11. ^{13}C NMR spectrum of (3*R*,3´*S*)-astaxanthin.

Calculated reaction mechanisms

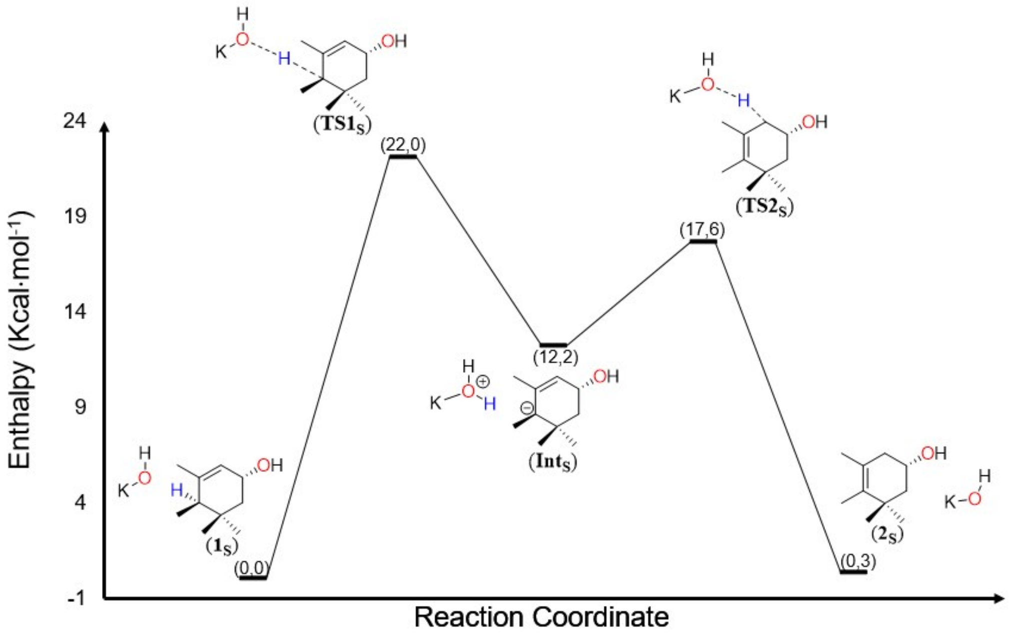

Figure S12. Energy profile for the conversion reaction of lutein (**1**) to zeaxanthin (**2**) calculated at the (PCM:*n*-butanol) M06-L/6-311+G(2d)//M06-L/6-31G(d) level, using sodium hydroxide as the base. Energy values are expressed as enthalpies in kcal·mol^{-1}.

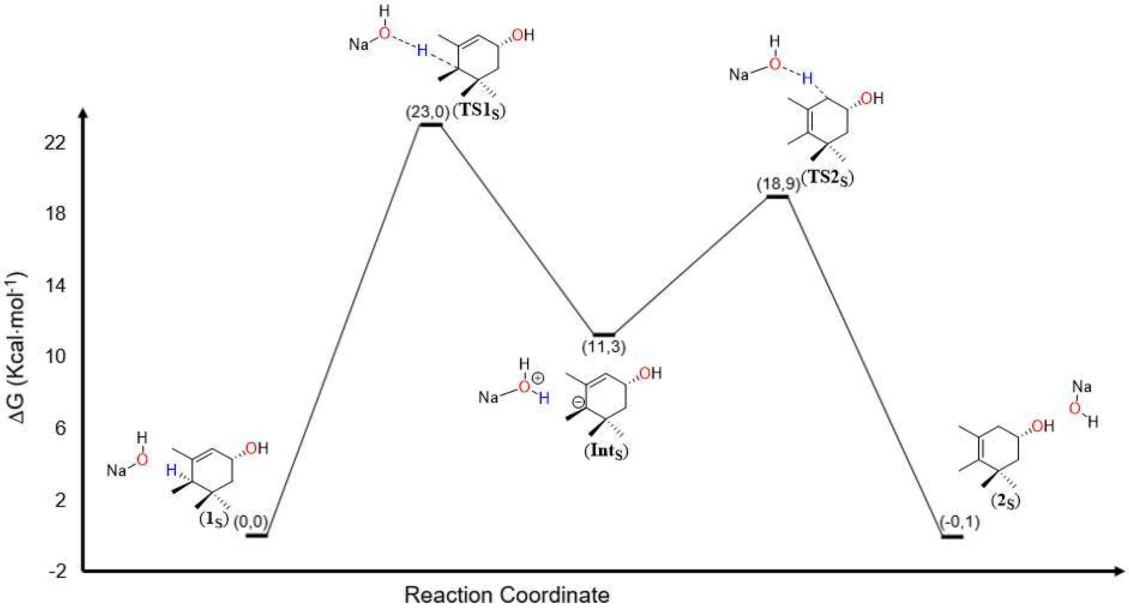

Figure S13. Energy profile for the conversion reaction of lutein (**1**) to zeaxanthin (**2**) calculated at the (PCM:*n*-butanol) M06-L/6-311+G(2d)//M06-L/6-31G(d) level, using sodium hydroxide as the base. Energy values are expressed as free energies in kcal·mol^{-1}.

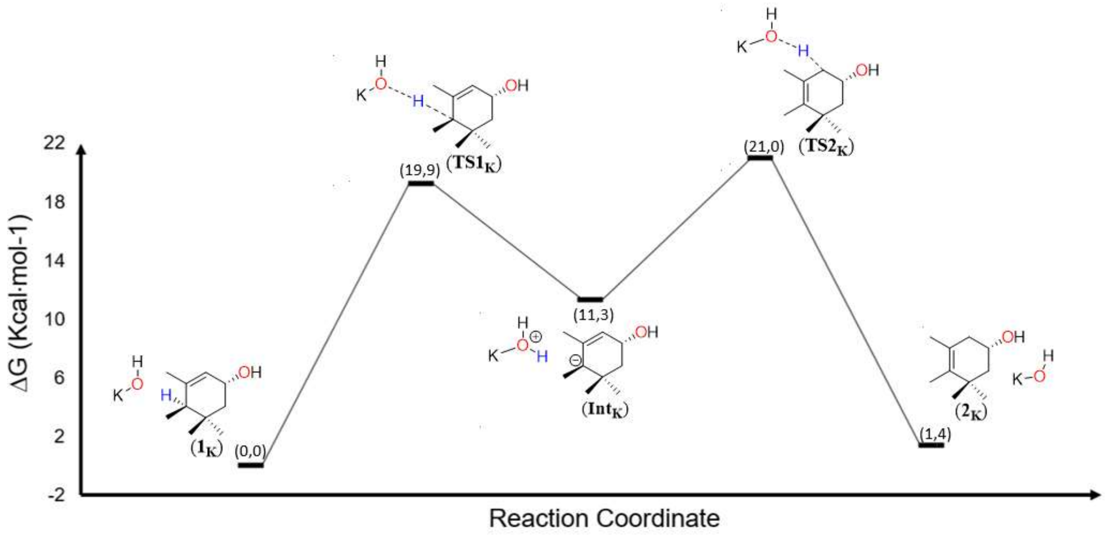

Figure S14. Energy profile for the conversion reaction of lutein (**1**) to zeaxanthin (**2**) calculated at the (PCM:*n*-butanol) M06-L/6-311+G(2d)//M06-L/6-31G(d) level, using potassium hydroxide as the base. Energy values are expressed as free energies in kcal·mol^{-1}.

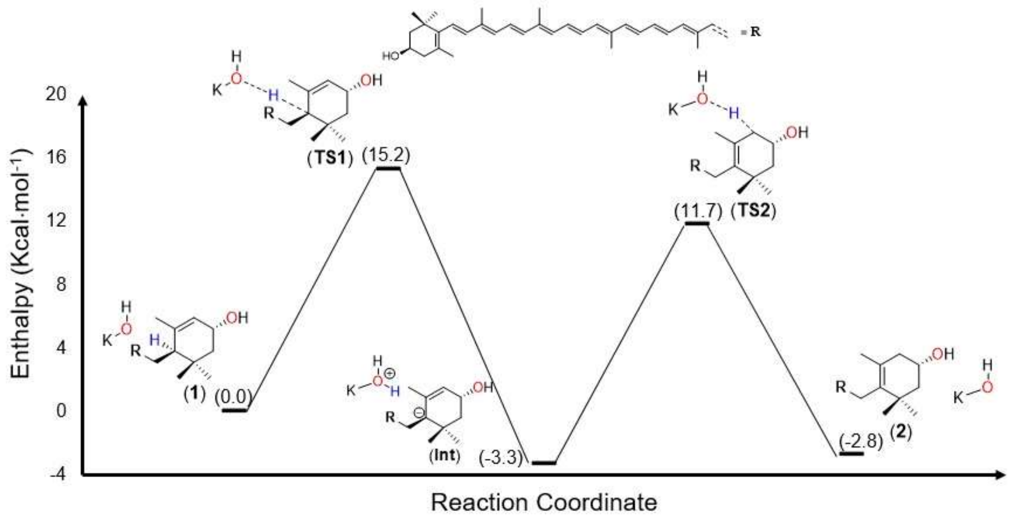

Figure S15. Energy profile for the conversion reaction of lutein (**1**) to zeaxanthin (**2**) calculated at the (PCM:*n*-butanol) M06-L/6-311+G(2d)//M06-L/6-31G(d) level, using potassium hydroxide as the base. Energy values are expressed as enthalpies in Kcal·mol^{-1}. **Note:** The real system is shown here for comparison with Figure 2.

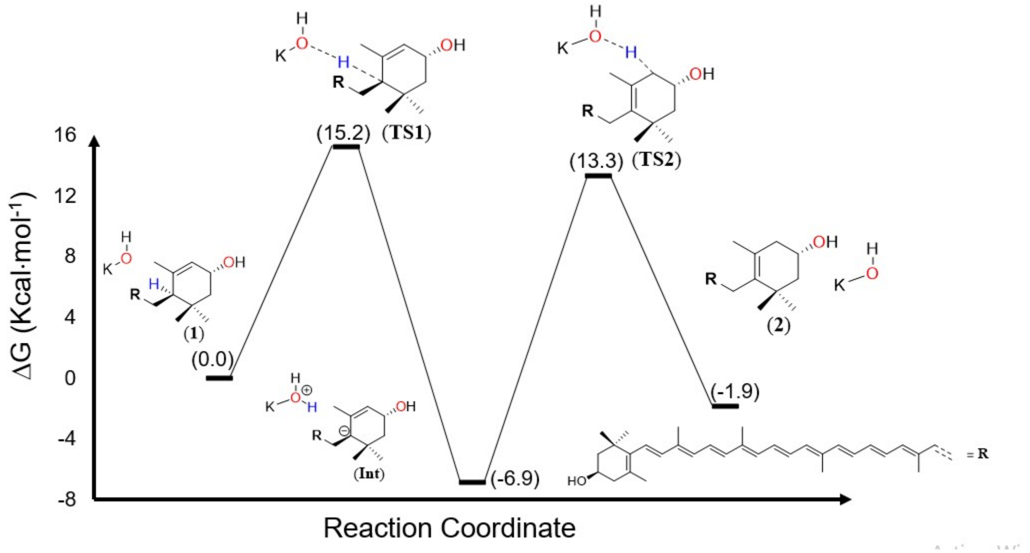

Figure S16. Energy profile for the conversion reaction from lutein (**1**) to zeaxanthin (**2**) with potassium hydroxide as base and calculated at the (PCM:*n*-butanol) M06-L/6-311+G(2d)//M06-L/6-31G(d) level. Energy values are expressed as free energies in Kcal·mol^{-1}. **Note:** The complete system is shown here for comparison with Figure S1.

Table S5. Cartesian coordinates (xyz) for the optimized geometries involved in the conversion reaction mechanism of lutein (**1**) to zeaxanthin (**2**), calculated at the (PCM:1-butanol)M06-L/6-311+G(2d)//M06-L/6-31G(d) level.

- With sodium hydroxide:

**1s**				**TS1s**			
E(scf) = –743.286248060 a.u.				E(scf) = –743.257407875a.u.			
				ν_{min} = –1188.20 cm^{-1}			
C	–2.288121000	0.018338000	–0.4511860	C	–2.01612500	0.568590000	0.878233000
C	–0.9063280	0.301398000	–1.052594000	C	–0.85050500	1.134301000	0.046747000
C	0.015676000	0.861882000	0.060124000	C	0.348123000	0.162257000	0.171328000
C	–0.040749000	0.020098000	1.319115000	C	–0.05275300	–1.24722500	–0.086716000
C	–1.076681000	–0.787234000	1.59470400	C	–1.330865000	–1.673380000	–0.00260300
C	–2.28306900	–0.951620000	0.729551000	C	–2.470405000	–0.82780400	0.455634000
H	–2.72304400	0.975336000	–0.115291000	H	–1.68524300	0.527794000	1.931006000
H	–2.96796800	–0.366484000	–1.229960000	H	–2.88597300	1.249775000	0.851181000
H	–1.05667200	–1.391862000	2.508188000	H	–1.58405600	–2.695882000	–0.305312000
C	1.129218000	0.161997000	2.239446000	C	1.000768000	–2.189619000	–0.60559200
H	1.055356000	–0.49652600	3.113911000	H	0.626275000	–3.21767800	–0.697694000
H	2.064280000	–0.06100400	1.698676000	H	1.339926000	–1.861581000	–1.600994000
H	1.231568000	1.198690000	2.596167000	H	1.901034000	–2.21795800	0.025152000
C	–1.04618800	1.313497000	–2.187275000	C	–0.529727000	2.536334000	0.56024000
H	–1.544294000	2.233141000	–1.85402100	H	–0.36367300	2.555229000	1.645205000
H	–0.06228300	1.597084000	–2.58689200	H	0.368337000	2.946712000	0.075199000
H	–1.635465000	0.891232000	–3.01284000	H	–1.36274700	3.219619000	0.343302000
C	–0.28075800	–0.972317000	–1.625102000	C	–1.25351900	1.270084000	–1.427229000
H	–0.954426000	–1.44839800	–2.350678000	H	-2.172032000	1.864808000	–1.53535800
H	0.660486000	–0.73436600	–2.141432000	H	–0.45310900	1.766065000	–1.992360000
H	–0.065103000	–1.711053000	–0.84493700	H	–1.442398000	0.293243000	–1.88551800
C	–0.275326000	2.292762000	0.419892000	C	1.237611000	0.380187000	1.306875000
H	–1.30965200	2.517952000	0.713418000	H	1.498083000	1.433769000	1.479097000

C	0.634895000	3.267543000	0.446092000
H	1.673493000	3.073612000	0.167423000
H	0.388036000	4.287198000	0.740561000
H	1.111036000	0.821773000	–0.32703200
O	–2.32528300	–2.330999000	0.322629000
H	–3.217072000	–0.73859500	1.367208000
H	–3.19893300	–2.455139000	–0.124273000
O	3.526371000	0.017561000	–0.56629200
H	3.438925000	0.462091000	0.296266000
Na	3.273421000	–2.11865800	–0.51106800

Ints

E(scf) = –743.274536505 a.u.

C	–2.334410000	0.591868000	0.61191700
C	–1.025215000	1.146533000	0.018076000
C	0.152322000	0.313854000	0.559538000
C	–0.100039000	–1.106738000	0.65366900
C	–1.337100000	–1.669540000	0.46829200
C	–2.589557000	–0.885537000	0.34140900
H	–2.298526000	0.741518000	1.71434800
H	–3.196594000	1.188976000	0.23896700
H	–1.428233000	–2.759651000	0.49403600
C	1.039160000	–2.049185000	0.93892900
H	0.737058000	–3.090856000	0.76601300
H	1.919065000	–1.836646000	0.31376000
H	1.385074000	–1.977383000	1.98047000
C	–0.962696000	2.628141000	0.39694200
H	–0.853449000	2.767000000	1.48194700
H	–0.132051000	3.154104000	–0.09308000
H	–1.888245000	3.133002000	0.08354900
C	–1.076667000	1.067541000	–1.51736200
H	–1.980516000	1.553632000	–1.91775200
H	–0.202141000	1.570272000	–1.95699300
H	–1.088340000	0.026574000	–1.86083300
C	1.355379000	0.954766000	0.927461000
H	1.332690000	2.031557000	0.720392000
C	2.585079000	0.562035000	1.455728000
H	2.769390000	–0.421998000	1.89167400
H	3.273156000	1.351290000	1.775524000
H	1.272971000	–0.123971000	–1.25159500
O	–3.181065000	–1.120867000	–0.96386200
H	–3.350313000	–1.255235000	1.11694900
H	–4.046645000	–0.643948000	–0.95222200
O	1.905646000	–0.384785000	–1.95980900
H	1.571812000	–1.261895000	–2.24324400
Na	3.833604000	–0.129414000	–0.73092100

2s

E(scf) = –743.295128858 a.u.

C	0.734695000	–1.815980000	–0.90817600
C	1.456339000	–0.914457000	0.10695100
C	1.007874000	0.547727000	–0.02294300
C	–0.086915000	0.917578000	–0.73548400
C	–0.934702000	–0.070475000	–1.49486900
C	–0.737615000	–1.519885000	–1.10555200
H	1.219627000	–1.693001000	–1.90352000
H	0.882471000	–2.880935000	–0.61957100
H	–0.732423000	0.044551000	–2.57852200
C	–0.592304000	2.324387000	–0.86020600
H	–1.687625000	2.323599000	–0.73466000

C	1.874492000	–0.508526000	2.11206600
H	1.667736000	–1.578789000	2.08226400
H	2.579594000	–0.172581000	2.87199700
H	1.257114000	0.504292000	–0.87036100
O	–3.443493000	-0.795606000	–0.60914200
H	–2.966180000	–1.331763000	1.36311500
H	–4.232249000	–0.330005000	–0.23704600
O	2.152121000	0.767931000	–1.682186000
H	1.899571000	0.238713000	–2.46057700
Na	3.690764000	–0.054870000	–0.29141600

TS2s

E(scf) = –743.257407875.u.

ν_{min} = – 1188.2010^{-1}

C	–2.016125000	0.568590000	0.878233000
C	–0.850505000	1.134301000	0.046747000
C	0.348123000	0.162257000	0.171328000
C	–0.052753000	–1.247225000	–0.08671600
C	–1.330865000	–1.673380000	–0.00260300
C	–2.470405000	–0.827804000	0.45563400
H	–1.685243000	0.527794000	1.93100600
H	–2.885973000	1.249775000	0.851181000
H	–1.584056000	–2.695882000	–0.30531200
C	1.000768000	–2.189619000	–0.60559200
H	0.626275000	–3.217678000	–0.69769400
H	1.339926000	–1.861581000	–1.60099400
H	1.901034000	–2.217958000	0.02515200
C	–0.529727000	2.536334000	0.560240000
H	–0.363673000	2.555229000	1.64520500
H	0.368337000	2.946712000	0.075199000
H	–1.362747000	3.219619000	0.34330200
C	–1.253519000	1.270084000	–1.42722900
H	–2.172032000	1.864808000	–1.53535800
H	–0.453109000	1.766065000	–1.99236000
H	–1.442398000	0.293243000	–1.88551800
C	1.237611000	0.380187000	1.306875000
H	1.498083000	1.433769000	1.479097000
C	1.874492000	–0.508526000	2.112066000
H	1.667736000	–1.578789000	2.08226400
H	2.579594000	–0.172581000	2.87199700
H	1.257114000	0.504292000	–0.87036100
O	–3.443493000	–0.795606000	–0.60914200
H	–2.966180000	–1.331763000	1.36311500
H	–4.232249000	–0.330005000	–0.23704600
O	2.152121000	0.767931000	–1.68218600
H	1.899571000	0.238713000	–2.46577000
Na	3.690764000	–0.054870000	–0.29141600

H	–0.155118000	3.010633000	–0.12851500
H	–0.390632000	2.737547000	–1.86124200
C	1.864410000	1.500237000	0.698736000
H	2.234990000	1.151916000	1.672879000
C	2.302831000	2.692011000	0.272149000
H	2.042354000	3.089705000	–0.70862500
H	2.958681000	3.306332000	0.887715000
C	2.960263000	–1.021911000	–0.18682300
H	3.201641000	–0.613405000	–1.17781000
H	3.563117000	–0.482401000	0.55565200
H	3.273124000	–2.075732000	–0.16972400
C	1.229443000	–1.411323000	1.54558200
H	1.535076000	–2.464093000	1.63387700
H	1.826988000	–0.838070000	2.26735400
H	0.176165000	–1.340431000	1.841893000
H	–2.000836000	0.199463000	–1.36527900
H	–1.138353000	–2.176459000	–1.91088600
O	–1.514850000	–1.745488000	0.08770800
O	–3.823656000	0.949499000	0.39436600
H	–3.899150000	0.274757000	–0.30575100
Na	–2.195236000	0.006892000	1.49723400
H	–1.280351000	–2.649315000	0.42236700

- With potassium hydroxide:

1_K

E(scf) = –1180.88449886 a.u.

C	–2.439383000	–0.272665000	–0.19458000
C	–1.235113000	0.276655000	–0.97024600
C	–0.264615000	0.957663000	0.031637000
C	0.003088000	0.094973000	1.250576000
C	–0.809224000	–0.914736000	1.60345400
C	–2.079436000	–1.273020000	0.90465900
H	–2.974352000	0.575084000	0.26692800
H	–3.155120000	–0.741768000	–0.89071600
H	–0.554654000	–1.523864000	2.47820800
C	1.214403000	0.463850000	2.048801000
H	1.424615000	–0.268560000	2.83929000
H	2.094249000	0.568329000	1.386407000
H	1.081131000	1.447150000	2.526080000
C	–1.712554000	1.286612000	–2.01148400
H	–2.294030000	2.097955000	–1.55560800
H	–0.862718000	1.745639000	–2.53631300
H	–2.347700000	0.798203000	–2.76313400
C	–0.51386700	–0.856161000	–1.70402400
H	–1.189787000	–1.345954000	–2.41798900
H	0.345091000	–0.466612000	–2.27108700
H	–0.171550000	-1.634783000	–1.01319200
C	–0.736797000	2.308620000	0.49302300
H	–1.740907000	2.339885000	0.93774500
C	–0.014268000	3.428076000	0.43975400
H	0.990855000	3.430252000	0.012288000
H	–0.388617000	4.379351000	0.81658200
H	0.762600000	1.113171000	–0.49608600
O	–1.940519000	–2.620686000	0.41935000
H	–2.926644000	–1.261931000	1.68548900
H	–2.835299000	–2.884835000	0.08935600
O	3.329273000	0.859795000	–0.53232700
H	3.032108000	1.698767000	–0.14208900
K	2.743542000	–1.525134000	–0.49461900

$TS1_K$

E(scf) = –1180.86344412 a.u.

ν_{min} = – 1333.3081 cm^{-1}

C	2.295470000	–0.492942000	0.88491100
C	1.126906000	–1.124172000	0.10882100
C	–0.090376000	–0.169986000	0.20681800
C	0.284774000	1.228784000	–0.14396400
C	1.556811000	1.679159000	–0.11791000
C	2.719043000	0.882925000	0.371588000
H	1.981571000	–0.393975000	1.93920200
H	3.176085000	–1.160794000	0.884629000
H	1.787671000	2.682785000	–0.49349100
C	–0.801766000	2.120452000	–0.68275100
H	–0.423294000	3.113981000	–0.95757400
H	–1.25069100	1.661639000	–1.578245000
H	–1.624083000	2.268391000	0.03299100
C	0.837627000	–2.500229000	0.70142600
H	0.683268000	–2.461540000	1.787701000
H	–0.060990000	–2.946636000	0.25089600
H	1.677968000	–3.182863000	0.511595000
C	1.504559000	–1.331836000	–1.36349400
H	2.425662000	–1.925036000	–1.45708900
H	0.695478000	–1.859995000	–1.88457800
H	1.678561000	–0.378159000	–1.87400800
C	–0.928762000	–0.314098000	1.39713300
H	–1.269530000	–1.365497000	1.63205200
C	–1.456320000	0.646337000	2.19849000
H	–1.186792000	1.699630000	2.10419900
H	–2.147080000	0.395560000	3.00403500
H	–0.96083000	–0.601223000	–0.80894100
O	3.676469000	0.804187000	–0.70447100
H	3.218899000	1.447064000	1.239938000
H	4.475347000	0.366083000	–0.32090800
O	–1.786550000	–0.969014000	–1.68068200
H	–1.361686000	–0.654317000	–2.49793800
K	–3.627510000	0.045640000	–0.19998100

Int_K

E(scf) = –1180.87785427 a.u.

C	2.696351000	0.610310000	–0.46638300
C	1.341857000	1.152243000	0.030559000
C	0.211585000	0.338164000	–0.62708800
C	0.471357000	–1.075842000	–0.74764300
C	1.689194000	–1.651095000	–0.47774700
C	2.927304000	–0.876429000	–0.22443100
H	2.755560000	0.797493000	–1.56216400
H	3.524138000	1.191772000	–0.00192500
H	1.781690000	–2.739542000	–0.54063800
C	–0.639332000	–2.004202000	–1.16370400
H	–0.340148000	–3.053206000	–1.03507000
H	–1.557922000	–1.834243000	–0.58319700
H	–0.917116000	–1.869321000	–2.21936900
C	1.313451000	2.643738000	–0.312298000
H	1.280258000	2.811427000	–1.39833700
H	0.453822000	3.162875000	0.133101000
H	2.217705000	3.134350000	0.077102000
C	1.264225000	1.033630000	1.562082000
H	2.123199000	1.520494000	2.051458000
H	0.347107000	1.512146000	1.936883000

$TS2_K$

E(scf) = –1180.86765647a.u.

ν_{min} = – 1513.1702^{-1}

C	–1.229004000	–1.829910000	0.61925000
C	–1.745727000	–0.551832000	–0.06255900
C	–0.902912000	0.668861000	0.34718100
C	0.186611000	0.518113000	1.185191000
C	0.883849000	–0.742020000	1.38791900
C	0.285216000	–1.939564000	0.69567700
H	–1.600714000	–1.856483000	1.65764800
H	–1.668834000	–2.708358000	0.11320100
H	1.121754000	–0.933897000	2.455107000
C	0.839098000	1.685072000	1.875566000
H	1.253257000	1.355926000	2.839622000
H	1.693159000	2.082937000	1.303249000
H	0.150241000	2.520150000	2.050382000
C	–1.355447000	1.938324000	–0.19194600
H	–2.38256700	1.928387000	–0.57791100
C	–0.699840000	3.109397000	–0.36929400
H	0.337478000	3.271417000	–0.08031800
H	–1.207171000	3.960695000	–0.82055900
C	–3.199778000	–0.36228900	0.405383000

H	1.260583000	–0.015408000	1.87954600
C	–0.966736000	0.995586000	–1.05800200
H	–0.953540000	2.066731000	–0.82331000
C	–2.150758000	0.628940000	–1.68411200
H	–2.347890000	–0.357487000	–2.10677200
H	–2.844209000	1.420857000	–1.98372300
H	–1.029886000	–0.232629000	1.05104400
O	3.416788000	–1.152858000	1.11675700
H	3.747338000	–1.222629000	–0.94766600
H	4.280245000	–0.677167000	1.18552500
O	–1.640850000	–0.560950000	1.74866900
H	–1.194688000	–1.373768000	2.06286000
K	–3.986565000	–0.068554000	0.57441300

2_K

E(scf) = –1180.89235949 a.u.

C	–1.251753000	–1.747781000	0.91526300
C	–1.795545000	–0.734692000	–0.10536500
C	–1.125070000	0.636972000	0.052926000
C	0.001380000	0.818022000	0.789135000
C	0.664130000	–0.299775000	1.552644000
C	0.246455000	–1.694568000	1.138235000
H	–1.730375000	–1.553638000	1.90211900
H	–1.565951000	–2.772042000	0.61245100
H	0.454267000	–0.165315000	2.63260300
C	0.743170000	2.115394000	0.91907500
H	1.825236000	1.916568000	0.81324500
H	0.435759000	2.864449000	0.18197900
H	0.599914000	2.562321000	1.91613600
C	–1.797003000	1.717977000	–0.68286100
H	–2.211592000	1.432962000	–1.65982000
C	–2.029175000	2.971572000	–0.27002900
H	–1.715197000	3.328688000	0.71068200
H	–2.563775000	3.683037000	–0.89853600
C	–3.305507000	–0.606928000	0.14370200
H	–3.510709000	–0.186414000	1.13795700
H	–3.789086000	0.039435000	–0.60095300
H	–3.783518000	–1.595781000	0.08975400
C	–1.602702000	–1.247628000	–1.54320000
H	–2.072672000	–2.235402000	–1.65874200
H	–2.072629000	–0.577420000	–2.27567900
H	–0.541738000	–1.346601000	–1.79715100
H	1.761326000	–0.194354000	1.44929900
H	0.522084000	–2.415541000	1.94188000
O	0.993884000	–2.022964000	–0.04713400
O	3.774373000	0.658529000	0.501475000
H	3.592124000	0.930174000	1.416309000
H	0.638211000	–2.883963000	–0.38399800
K	2.373262000	–0.078266000	–1.40112700

H	–3.244619000	–0.160468000	1.48347200
H	–3.705124000	0.461330000	–0.11415300
H	–3.782674000	–1.272932000	0.20763800
C	–1.785125000	–0.727820000	–1.58915800
H	–2.435819000	–1.570358000	–1.86638000
H	–2.183503000	0.170959000	–2.07954100
H	–0.793534000	–0.935905000	–2.00583100
H	2.139290000	–0.531376000	0.89724900
H	0.550796000	–2.864086000	1.24445500
O	0.865050000	–2.031724000	–0.63657900
O	3.303380000	–0.282440000	0.37390200
H	3.551838000	–1.18297500	0.097105000
H	0.377628000	–2.753572000	–1.10734000
K	1.622107000	0.470206000	–1.54815500

- With potassium hydroxide (Real system):

1

E(scf) = –2383.87582120 a.u.

C	–13.55839700	0.72053600	0.92315900
C	–14.62653200	–0.271854000	1.39764100
C	–15.19212000	–1.10692300	0.26857500
C	–14.08312000	–1.93101500	–0.34890100
C	–12.80779500	–1.17116000	–0.59182300
C	–12.54061400	0.02231000	0.00682700
H	–14.18123000	–0.96383500	2.14885100
H	–15.44026900	0.28710900	1.91257000
H	–13.87353000	–2.78702800	0.32778300
H	–14.42732100	–2.38983400	–1.2940680
C	–12.86412500	1.28175300	2.17117900
H	–12.15381200	2.08277100	1.92717200
H	–13.61235000	1.69983200	2.85959800
H	–12.31446900	0.49594100	2.70738100
C	–14.21384900	1.88776500	0.16919600
H	–14.91508200	2.42109000	0.82733500
H	–13.46539000	2.61638800	–0.17206200
H	–14.76853500	1.54398800	–0.71301100
C	–11.88038900	–1.85206300	–1.55326800
H	–11.28975100	–2.64228800	–1.0625020
H	–12.45464300	–2.35036400	–2.3474020
H	–11.16716200	–1.16363900	–2.0201340
C	–11.30594900	0.77110200	–0.1905480
H	–11.41884200	1.86104000	–0.18247000
C	–10.03925300	0.28378400	–0.27021300
H	–9.88220800	–0.79595400	–0.18936100
C	–8.84379400	1.07745800	–0.39352200
C	–7.62900600	0.4346690	–0.39008400
H	–7.65439200	–0.65855200	–0.30826600
C	–6.33514900	1.01759700	–0.46525100
H	–6.25977900	2.10575300	–0.54426700
C	–5.17748300	0.28463900	–0.43091300
H	–5.26547600	–0.80520200	–0.35057300
C	–8.97859500	2.56583900	–0.49799500
H	–9.60698900	2.84739100	–1.35536400
H	–9.47277700	2.98182600	0.39316400
H	–8.01477000	3.07210000	–0.60977300
C	–3.84997900	0.81060000	–0.47940100
C	–2.78855400	–0.06963900	–0.42031100
H	–3.04162600	–1.13393200	–0.34532200
C	–1.40704500	0.23616500	–0.43667500
H	–1.09909300	1.28470800	–0.50749800
C	–0.41530700	–0.71273900	–0.35890100
H	–0.72189900	–1.76163100	–0.28631400
C	–3.67007600	2.29532500	–0.58610900
H	–4.13249600	2.68443400	–1.50510100
H	–4.16197600	2.81458300	0.24914600
H	–2.61810400	2.59641600	–0.59071400
C	0.96573400	–0.40330100	–0.36128800
H	1.21603200	0.66149800	–0.43803400
C	2.02902900	–1.27761800	–0.26930300
C	3.35499200	–0.74282200	–0.27603600
H	3.43795800	0.34623000	–0.37106400
C	4.51468600	–1.46214300	–0.16515500
H	4.44756400	–2.54914600	–0.06176300
C	1.85452500	–2.76205500	–0.15368900

TS1

E(scf) = –2383.85609406 a.u.

ν_{min} = –1670.2610cm^{-1}

C	–13.78647100	0.80383700	0.68569400
C	–14.87162500	–0.12715100	1.2409470
C	–15.33625500	–1.15250700	0.2287040
C	–14.17052900	–2.03494300	–0.1592490
C	–12.89842300	–1.2852610	–0.4478410
C	–12.69507500	–0.00145100	–0.0404110
H	–14.47430600	–0.67339100	2.1269620
H	–15.73060700	0.48555900	1.59634200
H	–13.99409100	–2.75497400	0.6683220
H	–14.4382040	–2.65979400	–1.0312560
C	–13.18921900	1.57427300	1.87042400
H	–12.46643900	2.33618100	1.54923400
H	–13.98780200	2.08644100	2.42603200
H	–12.67552200	0.89661100	2.56621700
C	–14.40651000	1.81849900	–0.2874540
H	–15.16106200	2.43036700	0.22790700
H	–13.64844300	2.50321200	–0.6922600
H	–14.89310300	1.32202200	–1.1368930
C	–11.89647300	–2.08327000	–1.2280540
H	–11.28166800	–2.7261260	–0.5777020
H	–12.4090360	–2.75800800	–1.9282260
H	–11.2030190	–1.453034000	–1.796793
C	–11.4705410	0.74754700	–0.2903270
H	–11.61103000	1.82381000	–0.4386620
C	–10.18738200	0.29500700	–0.2650430
H	–10.00131100	–0.7574410	–0.0316310
C	–9.01113000	1.09882400	–0.46867700
C	–7.77814800	0.4974650	–0.36472200
H	–7.77789900	–0.5754500	–0.1363150
C	–6.4981990	1.09466500	–0.51032900
H	–6.44747200	2.16293100	–0.7388930
C	–5.32244700	0.39987900	–0.37184000
H	–5.38956300	–0.67050500	–0.1421110
C	–9.18159800	2.55693900	–0.77000800
H	–9.78614100	2.70970400	–1.67615500
H	–9.71667700	3.06990600	0.04372700
H	–8.22733400	3.07305200	–0.91543000
C	–4.00729700	0.93684600	–0.49063500
C	–2.92589100	0.09382500	–0.31031800
H	–3.16131300	–0.95413500	–0.0874520
C	–1.55213400	0.4140940	–0.37113500
H	–1.25952200	1.44692800	–0.5874380
C	–0.54365100	–0.50465600	–0.1677430
H	–0.8430790	–1.53609500	0.0481050
C	–3.8522120	2.39609000	–0.8016110
H	–4.31950800	2.65013300	–1.76468700
H	–4.35188800	3.01886800	–0.0449080
H	–2.80449800	2.70912900	–0.84934100
C	0.83118000	–0.19389400	–0.2140800
H	1.07836600	0.85251200	–0.42960000
C	1.90461900	–1.04759400	–0.01358800
C	3.22514100	–0.52552200	–0.08963300
H	3.31279300	0.54446300	–0.31252400
C	4.39017200	–1.23604000	0.09832300
H	4.30754100	–2.30349500	0.3245320

H	2.28706600	–3.13930000	0.78462300
H	0.80470400	–3.06956300	–0.18274700
H	2.37885200	–3.28587300	–0.96600100
C	5.80548500	–0.86245700	–0.16881200
H	5.82392100	0.22787100	–0.28558200
C	7.02128700	–1.48236600	–0.03133800
C	8.21392300	–0.66772000	–0.05243600
H	8.06941400	0.41132900	–0.20953900
C	9.48234900	–1.10142000	0.11438600
C	7.17545100	–2.96085800	0.14741200
H	7.64717900	–3.19336400	1.11396900
H	6.22307300	–3.49884500	0.10293700
H	7.83822900	–3.38192800	–0.62317600
O	–16.19478500	–2.01671300	0.71852400
H	–16.92356800	–1.46317500	1.09472600
H	–15.62065800	–0.43287900	–0.5129610
C	12.56753500	–1.78476200	–0.24930500
C	11.79328300	–0.61345900	–0.86675000
C	10.671049000	–0.18668200	0.11541100
C	11.18622700	–0.03197800	1.53384800
C	12.32913400	–0.600499000	1.94781700
C	13.19800400	–1.47840500	1.10822800
H	11.87241500	–2.63374500	–0.12884900
H	13.35224900	–2.12987800	–0.94380900
H	12.672366000	–0.42516000	2.97362400
C	10.32318900	0.78384200	2.44477500
H	10.82958300	1.02758100	3.38732300
H	10.00080800	1.70987100	1.94026200
H	9.39291700	0.24728700	2.69107200
C	11.19564500	–1.04821800	–2.20308700
H	10.57060700	–1.94491600	–2.09959800
H	10.56557500	–0.25531400	–2.63100200
H	11.98974200	–1.27404200	–2.92831700
C	12.71379500	0.58181300	–1.12193800
H	13.57073100	0.29332000	–1.74594000
H	12.16574800	1.37556600	–1.65014700
H	13.11279500	0.99651900	–0.19001600
O	14.48118800	–0.83711400	0.99857800
H	15.07928900	–1.49950600	0.57226200
O	8.74160900	2.84339000	0.1337150
H	8.34562300	2.23977700	0.78383100
H	10.27337300	0.85381700	–0.20618400
K	10.61129300	4.30155300	–0.47890800
H	9.67803600	–2.16676300	0.29648600
H	13.34454300	–2.47463500	1.66513200

Int

E(scf) = –2383.88980483 a.u.

C	13.75578300	0.10640400	–0.5825390
C	14.77327800	–1.02108700	–0.36522200
C	15.01547600	–1.31521000	1.09987000
C	13.72378900	–1.78340900	1.73094800
C	12.53259700	–0.93074100	1.38260500
C	12.51749500	–0.08961600	0.30981100
H	14.40197100	–1.95480900	–0.8467800
H	15.72814800	–0.75331000	–0.87135100
H	13.54449100	–2.83152300	1.40868100
H	13.8341600	–1.83504100	2.82979300
C	13.36088700	0.09155600	–2.06472100
H	12.71212200	0.93653800	–2.33003200

C	1.72279500	–2.50661400	0.2880880
H	2.15263600	–2.76654700	1.26644900
H	0.67158000	–2.81030200	0.29701500
H	2.24329200	–3.13150000	–0.45201200
C	5.68190500	–0.66989000	0.01970700
H	5.72149600	0.40162600	–0.21078900
C	6.90112400	–1.29991900	0.20430300
C	8.10216000	–0.53272800	0.10689100
H	7.96928800	0.52899800	–0.13356900
C	9.37188200	–0.9995500	0.33609800
C	7.0022670	–2.76276600	0.5239400
H	7.5101030	–2.92245500	1.48674600
H	6.0267800	–3.25561100	0.5794500
H	7.60309300	–3.29316600	–0.22873200
O	–16.35781100	–2.00218400	0.7492600
H	–17.12071800	–1.41374300	0.9737510
H	–15.71314700	–0.6300990	–0.6843490
C	11.26482800	0.7029900	2.36872600
C	11.61753500	–0.4186700	1.37763400
C	10.6332110	–0.29858400	0.17827000
C	10.54968800	1.09738200	–0.35093300
C	10.95702400	2.16762400	0.3605110
C	11.3794710	2.11334700	1.79035300
H	10.22390200	0.5424300	2.69934900
H	11.89604300	0.6332270	3.27217700
H	10.97085500	3.15801400	–0.10882600
C	10.08832800	1.2652510	–1.7736800
H	10.28840900	2.27758400	–2.14950200
H	10.59464700	0.53397300	–2.42139600
H	9.0127400	1.0752220	–1.89403500
C	11.50254100	–1.76417300	2.0905200
H	10.53030500	–1.89262200	2.58342700
H	11.6381470	–2.60056100	1.38961400
H	12.27887400	–1.8499190	2.86312300
C	13.06861200	–0.27399500	0.90772900
H	13.75556900	–0.25914700	1.76504900
H	13.34689100	–1.11626000	0.26088800
H	13.21996700	0.66836500	0.36926400
O	12.715460	2.64787200	1.87354500
H	12.91445000	2.72854400	2.8389250
O	11.93187000	–1.51695700	–1.7967680
H	11.19046700	–1.7112240	–2.39905200
H	11.24636400	–0.96089300	–0.7942000
K	13.63402600	0.30796200	–2.44309400
H	9.4585900	–2.04759800	0.64383300
H	10.6889280	2.80193400	2.40151200

TS2

E(scf) = –2383.86656597.u.

ν_{min} = –1681.6380 cm^{-1}

C	–13.27344500	–0.38955200	0.07664100
C	–11.98683300	–1.1786590	0.3788130
C	–10.77398000	–0.58230000	–0.3627420
C	–10.94184100	0.52058000	–1.1947490
C	–12.04713800	1.44698900	–1.0717410
C	–13.08020200	1.11375100	–0.0239840
H	–13.6853090	–0.72577700	–0.9009260
H	–14.03976900	–0.64396300	0.8437500
H	–12.48775400	1.72934400	–2.043750
C	–9.93617200	0.91930300	–2.23676200
H	–9.27347300	1.72041200	–1.86990500

H	14.2605800	0.15193200	–2.69416100	H	–9.30353500	0.0893120	–2.5702980
H	12.82881100	–0.83407800	–2.32464500	H	–10.45789100	1.34083400	–3.1087560
C	14.40237100	1.46554900	–0.27032000	C	–12.22563500	–2.61840500	–0.1091590
H	15.26278800	1.64084000	–0.93249800	H	–12.34763200	–2.6512060	–1.2003860
H	13.69476500	2.29206800	–0.42388700	H	–11.40373900	–3.29459700	0.1615950
H	14.75547400	1.51946600	0.76754600	H	–13.14188100	–3.02300800	0.3453070
C	11.38511800	–1.0903520	2.33557400	C	–11.7352200	–1.24735000	1.89350600
H	10.75481400	–1.9607410	2.0908200	H	–12.59004900	–1.71543700	2.4043830
H	11.75835300	–1.26246400	3.35565700	H	–10.84468700	–1.85155700	2.1181190
H	10.72367500	–0.21615300	2.34915500	H	–11.59348100	–0.25287200	2.3327100
C	11.3722800	0.73049000	–0.06599900	H	–11.45601800	2.59081900	–0.7619150
H	11.61991800	1.6999940	–0.5125300	H	–14.05064700	1.59290400	–0.2844930
C	10.05206100	0.3934950	–0.01515600	O	–12.62905200	1.66579800	1.24039400
H	9.78291100	–0.6075970	0.33610600	O	–10.76434000	3.68191800	–0.4128250
C	8.94782700	1.21548500	–0.42036600	H	–10.28909000	3.86073200	–1.2435680
C	7.67175000	0.69888600	–0.3311980	H	–13.24203500	1.31640500	1.93482200
H	7.59942600	–0.32814600	0.05010400	C	–9.53529700	–1.27528800	–0.1789580
C	6.43615900	1.30864300	–0.65524200	C	–8.24581600	–0.82470600	–0.3625360
H	6.43969700	2.33413600	–1.03632900	H	–8.07934300	0.20576900	–0.6880040
C	5.22295400	0.66686500	–0.50534000	C	–7.06149100	–1.58239200	–0.1245770
H	5.24996700	–0.36048400	–0.11963200	C	–5.83128600	–0.96794700	–0.2944360
C	9.23062200	2.59932300	–0.92228700	H	–5.85528900	0.08182000	-0.61155100
H	9.77246300	3.19561700	–0.17218500	C	–4.5462260	–1.52217400	–0.10641700
H	9.87646900	2.57837600	–1.81434800	H	–4.47309600	–2.57014900	0.19936100
H	8.31944300	3.14623300	–1.18709600	C	–3.37826000	–0.8134900	–0.28346600
C	3.93838600	1.18784500	–0.79313900	H	–3.46484200	0.23761300	–0.58354100
C	2.82080500	0.38788400	–0.56832400	C	–7.18592500	–3.01353600	0.30817800
H	3.03055000	–0.61584600	–0.17568600	H	–7.72870000	–3.09458700	1.26177100
C	1.46566400	0.68363100	–0.76973700	H	–7.76068900	–3.60143400	–0.4217000
H	1.18589100	1.66894400	–1.15823800	H	–6.21536400	–3.50177400	0.43917900
C	0.43900400	–0.21616900	–0.49362900	C	–2.05765300	–1.3102590	–0.1093580
H	0.74769200	–1.19318700	–0.10377600	C	–0.98910300	–0.45053700	–0.3082890
C	3.82746100	2.58637200	–1.32726500	H	–1.24221700	0.57790800	–0.5924880
H	4.25187600	3.32175500	–0.62653900	C	0.38714800	–0.73230500	–0.18300700
H	4.38828300	2.70443400	–2.26710900	H	0.69499600	–1.74661100	0.09367000
H	2.79047300	2.87939600	–1.52370900	C	1.38458300	0.19865200	–0.38439100
C	–0.92416500	0.03070200	–0.66654400	H	1.07959000	1.21473200	–0.65629900
H	–1.19271900	1.02092900	–1.05509400	C	–1.87157600	–2.74439500	0.29089100
C	–2.00034100	–0.82829700	–0.3915710	H	–2.32464000	–2.94483000	1.27296800
C	–3.31542900	–0.39111600	–0.6090040	H	–2.36660000	–3.42170000	–0.4197750
H	–3.43781000	0.62500000	–1.00309900	H	–0.81801400	–3.03501400	0.34759600
C	–4.48098500	–1.12279700	–0.3568580	C	2.76125500	–0.08757500	–0.25665200
H	–4.35703900	–2.13433000	0.04202500	H	3.01067600	–1.12125800	0.01224000
C	–1.77326600	–2.21452900	0.14744200	C	3.82974000	0.77353500	–0.42788600
H	–2.26910200	–2.97144300	–0.4766270	C	5.15102900	0.26832900	–0.25050500
H	–0.71308400	–2.47976700	0.20265700	H	5.23203600	–0.79370600	0.01088300
H	–2.19370500	–2.32662900	1.15774800	C	6.31669900	0.98164300	–0.37713400
C	–5.77282800	–0.65379900	–0.5631990	H	6.25380400	2.04266100	–0.63465200
H	–5.86268800	0.36725900	–0.95542300	C	3.65517200	2.21812600	–0.79132300
C	–6.99598500	–1.31913700	–0.3169690	H	2.60537300	2.49632100	–0.92772100
C	–8.19810200	–0.65087700	–0.5564120	H	4.07638700	2.87687700	–0.01734100
H	–8.09772800	0.35987800	–0.94757300	H	4.18797200	2.46078200	–1.72259100
C	–9.47827300	–1.21940300	–0.3461040	C	7.60268500	0.40818600	–0.19452500
C	–7.00730300	–2.72789600	0.21322900	H	7.61457500	–0.65976700	0.05526100
H	–7.60196500	–3.39268800	–0.4292640	C	8.82830000	1.02448500	–0.29790700
H	–6.00381500	–3.15817400	0.28621300	C	10.01239200	0.23685700	–0.07793300
H	–7.46151400	–2.7814800	1.21328400	H	9.83567000	–0.81308800	0.17317500
O	15.98518300	–2.34455100	1.29200300	C	11.29191100	0.6984010	–0.11740300
H	16.82420700	–2.01383400	0.88553300	H	11.42419100	1.77288400	–0.28353900
H	15.35141000	–0.38251400	1.61574100	C	8.98254200	2.47895400	–0.62346500

C	–13.14172200	–1.38007300	–1.0359450
C	–11.92868300	–1.72756400	–0.1575200
C	–10.77733000	–0.75272200	–0.4647360
C	–11.16757600	0.6232400	–0.7317930
C	–12.47076300	0.99610800	–0.8787750
C	–13.62093700	0.05864400	–0.9337140
H	–12.86544300	–1.5769980	–2.0963280
H	–13.98109200	–2.0694270	–0.7953780
H	–12.69773100	2.0601200	–1.0004450
C	–10.13531200	1.71478700	–0.8164410
H	–10.62112200	2.70022900	–0.8374700
H	–9.43731500	1.68708700	0.03154800
H	–9.5217190	1.63524700	–1.7250320
C	–11.58186500	–3.19096200	–0.4519040
H	–11.18825400	–3.3169940	–1.4701340
H	–10.84389800	–3.60438200	0.2480640
H	–12.48690300	–3.8078310	–0.3603950
C	–12.29625200	–1.62275900	1.3310080
H	–13.15743100	–2.26512400	1.5695080
H	–11.45029200	–1.94258700	1.9572530
H	–12.56701600	–0.59819300	1.6081460
O	–14.45808400	0.30502400	0.2167030
H	–15.26393300	–0.25423200	0.0870400
O	–10.78810600	1.42585600	2.4364410
H	–11.55468600	1.36273100	1.8269210
K	–9.55809300	3.81027800	2.1981170
H	–9.42028200	–2.26170900	–0.0279230
H	–10.25852800	0.63737700	2.1932120
H	–14.24011500	0.29584700	–1.8725130

2

E(scf) = –2383.88567346 a.u.

C	–13.29884700	–0.36503000	0.0722740
C	–12.01503000	–1.17088700	0.3426670
C	–10.80820700	–0.59958800	–0.4202900
C	–10.91086800	0.51565000	–1.1989300
C	–12.12136800	1.40552800	–1.1845070
C	–13.09466500	1.13058500	–0.0589810
H	–13.74979200	–0.7121390	–0.8848640
H	–14.04208300	–0.59304700	0.8684770
H	–12.64941900	1.34232400	–2.1561930
C	–9.85777400	1.03082700	–2.1271690
H	–9.38602700	1.92958600	–1.6805360
H	–9.08938700	0.29371600	–2.3812090
H	–10.33491000	1.37427300	–3.0590330
C	–12.29450100	–2.60882000	–0.1275890
H	–12.4150780	–2.65494300	–1.2186070
H	–11.50077900	–3.31081400	0.1594090
H	–13.22694500	–2.97064600	0.3289840
C	–11.71116900	–1.22598700	1.8480500
H	–12.55312800	–1.67713900	2.3930070
H	–10.81969300	–1.83752000	2.0475160
H	–11.5398040	–0.22632700	2.2655140
H	–11.75280400	2.45092800	–1.1142570
H	–14.06977900	1.62031600	–0.2830910
O	–12.54726800	1.71860000	1.1350190
O	–9.48402000	3.77416000	–0.4308020
H	–9.92706800	3.92154400	–1.2832340
H	–13.13804100	1.44798100	1.8831490
C	–9.57391600	–1.35659800	–0.2605660

H	8.02239700	2.98385900	–0.76937000
H	9.51867800	3.00859000	0.17864000
H	9.579339000	2.62322600	–1.5360830
C	12.52009000	–0.04342100	0.1390490
C	13.61293200	0.77718200	0.84333200
C	13.97208600	–2.09492800	0.08857200
C	14.74416800	–0.11936800	1.37321300
C	15.17387600	–1.23582200	0.44781900
H	15.61629700	0.51697700	1.6394000
H	14.40043600	–0.59971400	2.31608200
C	14.16077900	1.83816700	–0.12534100
H	13.37628200	2.54453100	–0.43051400
H	14.95293600	2.42237400	0.36497800
H	14.58442800	1.37811000	–1.02298400
C	13.02110200	1.50908100	2.05734000
H	12.26527200	2.24958700	1.76305900
H	12.54851600	0.80527800	2.75660700
H	13.81555200	2.04116700	2.60048600
C	12.71660300	–1.33527900	–0.2430990
H	13.75762800	–2.78784900	0.92906300
C	11.71881900	–2.14193500	–1.0199200
H	11.10618800	–2.78555200	–0.3680340
H	11.02402500	–1.52000900	–1.5957940
H	12.23777000	–2.81864300	–1.7140550
H	14.23676700	–2.75619000	–0.7586140
H	–9.62460400	–2.28288000	0.24083500
K	–9.93609700	2.07637000	1.46336400
O	15.77955700	–0.66390200	–0.7145440
H	16.03068300	–1.42176900	–1.2984500
H	15.92356200	–1.87201900	0.97493500

C	–8.30029800	–0.87311900	–0.2524750
H	–8.13477700	0.20138900	–0.3828530
C	–7.10667600	–1.65573200	–0.0727100
C	–5.89275500	–1.00957800	–0.1073940
H	–5.92183100	0.07577800	–0.2630430
C	–4.59791400	–1.58005400	0.0117050
H	–4.51733800	–2.66041600	0.1625720
C	–3.44421000	–0.84337800	–0.0701050
H	–3.54015300	0.23777100	–0.2253070
C	–7.23747200	–3.13450400	0.1286680
H	–7.84943500	–3.36064900	1.0146770
H	–7.74693900	–3.60831300	–0.7232150
H	–6.27079700	–3.63126400	0.2573920
C	–2.11374100	–1.35376200	0.0167140
C	–1.05954800	–0.47020400	–0.1054120
H	–1.32191400	0.58309700	–0.2607500
C	0.32383000	–0.76073600	–0.0619360
H	0.64178400	–1.79737500	0.0920590
C	1.30634100	0.19118300	–0.2074600
H	0.98809100	1.22744900	–0.3632580
C	–1.92023200	–2.82541700	0.22891600
H	–2.37941900	–3.15337000	1.17291700
H	–2.40675100	–3.40617600	–0.5681370
H	–0.86531700	–3.11508900	0.25424200
C	2.68995200	–0.09939000	–0.16978500
H	2.95319700	–1.15166400	–0.00902600
C	3.74304100	0.78218800	–0.31210200
C	5.07463800	0.27139100	–0.24027100
H	5.17197800	–0.80718100	–0.06877000
C	6.22674400	1.00377500	–0.36365500
H	6.14418900	2.08062200	–0.53714100
C	3.54787000	2.25145500	–0.53826600
H	2.49303500	2.54220200	–0.55040000
H	4.04778600	2.84078200	0.24405600
H	3.99228200	2.56779600	–1.49325600
C	7.52426400	0.43294900	–0.27057800
H	7.55623300	-0.64804600	–0.08872000
C	8.73685400	1.07263300	–0.37486700
C	9.93662800	0.29109400	–0.22330900
H	9.78088500	–0.77255000	–0.02139500
C	11.20629300	0.77768900	–0.25813900
H	11.31699700	1.86137600	–0.37488300
C	8.86297300	2.54445200	–0.62376900
H	7.89341800	3.0403380	–0.73235400
H	9.40099600	3.03971100	0.1986490
H	9.44567000	2.74494800	–1.5346590
C	12.45232200	0.0520350	–0.0528630
C	13.53965000	0.86244300	0.6730000
C	13.97097100	–1.94089100	–0.2524490
C	14.64862100	–0.05749400	1.19967900
C	15.12992400	–1.05183200	0.16452200
H	15.49841000	0.56143500	1.56215800
H	14.26353400	–0.62794500	2.07773000
C	14.13051300	1.89960000	–0.29414300
H	13.35471100	2.57356300	–0.68323000
H	14.87864600	2.51972300	0.22048800
H	14.61734100	1.42235500	–1.15447200
C	12.94734000	1.60609900	1.87723800
H	12.21398600	2.36645300	1.57735500
H	12.44834300	0.91179400	2.5673790

H	13.74714200	2.11681800	2.43213100
C	12.67726800	–1.21379200	–0.5028080
H	13.80622500	–2.69573900	0.54780200
C	11.68331300	–2.01193100	–1.2930470
H	11.09363800	–2.68736800	–0.6526240
H	10.96715200	–1.38363100	–1.8352660
H	12.20219800	–2.65407900	–2.0185710
O	16.24231600	–1.81977600	0.62141000
H	15.51431700	–0.50991600	–0.7271350
H	15.93164700	–2.29504700	1.43347500
H	14.24540800	–2.53024900	–1.1468610
H	–9.69378500	–2.42336400	–0.0479190
K	–9.86606400	2.16552400	1.41741900

Table S6. Oxidation reaction mechanism enthalpy and free energy values (Scheme S1).

**Pathway 1**	ΔH	ΔG	**Pathway 2**	ΔH	ΔG
**I**	2,45	–3,91	**I**	2,45	–3,91
**II**	–36,12	–29,67	**II**	–36,12	–29,67
**III$_1$**	1,28	–3,54	**III**	–9,11	–13,94
**IV$_1$**	–35,31	–28,08	**IV**	–16,37	–20,60
**V$_1$**	–10,79	–16,16	**V**	0,72	–4,76
**VI$_1$**	–14,69	–19,59	**VI**	–34,75	–28,07
**VII**	–11,60	–17,03	**VII**	–11,60	–17,03
**3**	–18,48	–23,18	**3**	–18,48	–23,18
**Total =**	–123,26	–141,16	**Total =**	–123,26	–141,16

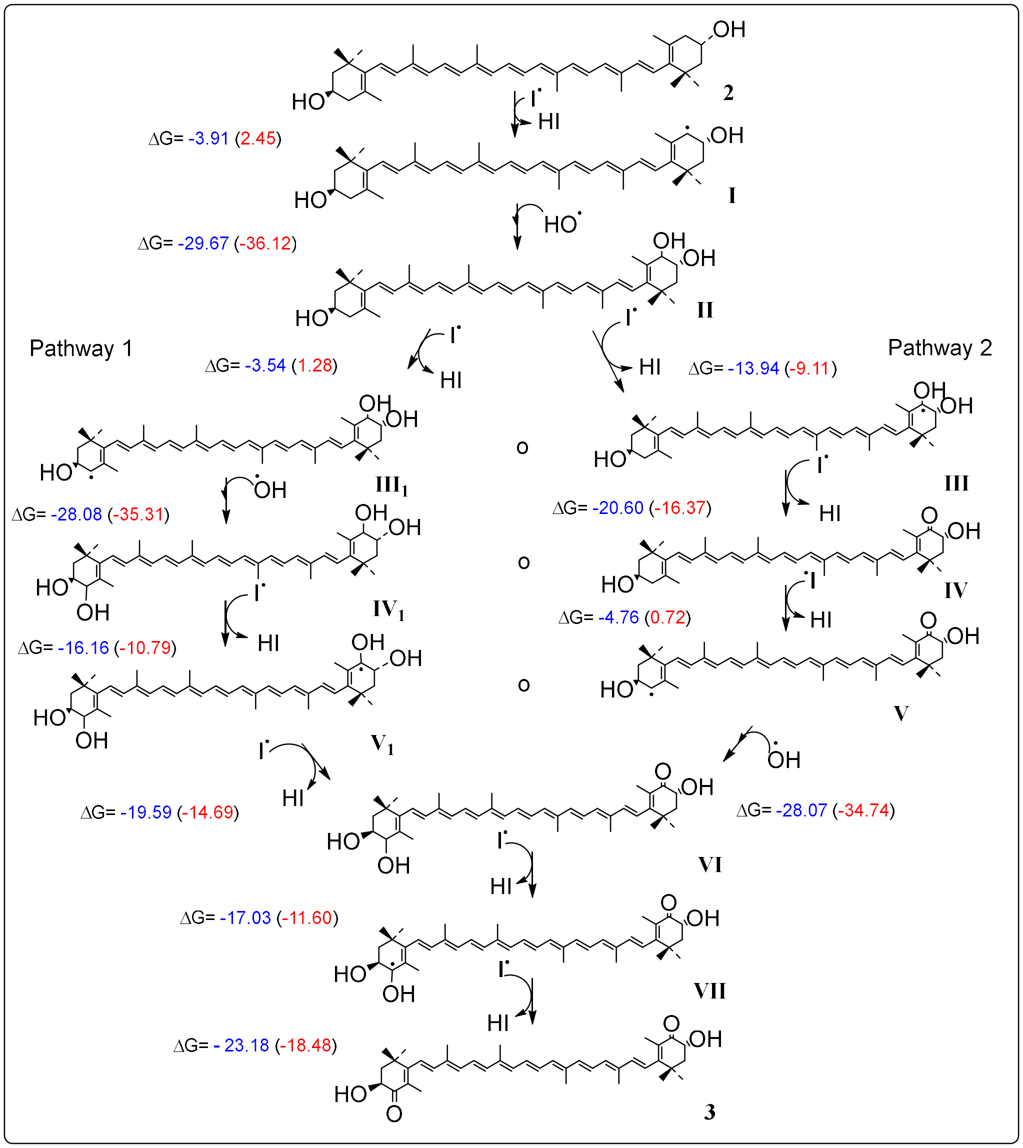

Scheme S1. Possible pathways for the allylic oxidation of **2** to **3**.

Table S7. Cartesian coordinates (xyz) for the optimized geometries involved in the oxidation reaction mechanism calculated at the (PCM:1-butanol)M06-L/6-311+G(2d)//M06-L/6-31G(d) level.

2

E(scf) = –1708.05727324 a.u.

C	14.065214000	–0.457466000	0.520507000
C	12.78997600	0.354519000	0.796002000
C	11.67304100	0.004001000	–0.195403000
C	11.78000400	–1.032307000	–1.068227000
C	12.95014600	–1.973330000	–1.081155000
C	13.83242200	–1.909352000	0.146241000
H	14.61454900	0.008322000	–0.312911000
H	14.73196600	–0.382906000	1.395057000
H	13.56073900	–1.784514000	–1.980270000
C	10.78866500	–1.334764000	–2.149322000
H	10.12728200	–2.171486000	–1.879010000
H	10.15230300	–0.480955000	–2.399883000
H	11.31514600	–1.653158000	–3.060989000
C	13.18545000	1.835104000	0.679111000
H	13.42021600	2.109364000	–0.357352000
H	12.40349400	2.511417000	1.042889000
H	14.07941900	2.028373000	1.287001000
C	12.28158200	0.109827000	2.224908000
H	13.06378300	0.344762000	2.961344000
H	11.41780400	0.750911000	2.442719000
H	11.97040200	–0.929969000	2.361738000
H	12.58297800	–3.004618000	–1.195753000
H	14.80516100	–2.38129800	–0.085371000
O	13.17744400	–2.65648600	1.167228000
H	13.68368200	–2.53208300	1.982581000
C	10.48956600	0.84953900	–0.107928000
C	9.19572100	0.44880900	–0.215161000
H	8.99059200	–0.61187900	–0.375668000
C	8.03944900	1.29490100	–0.097344000
C	6.79523200	0.72104500	–0.189438000
H	6.77054100	–0.36123400	–0.349412000
C	5.52796300	1.35372500	–0.102582000
H	5.49628300	2.43376300	0.050656000
C	4.34241500	0.67542600	–0.200411000
H	4.39161300	–0.40732100	–0.349248000
C	8.24643200	2.76060100	0.127775000
H	8.78849700	2.94339400	1.066166000
H	8.85597700	3.20235200	–0.671751000
H	7.30867300	3.31981700	0.176678000
C	3.03498100	1.24066800	–0.123723000
C	1.94571100	0.40210500	–0.231914000
H	2.16588000	–0.66033000	–0.374217000
C	0.57442100	0.73861000	–0.179964000
H	0.29186400	1.78473000	–0.040020000
C	–0.43819000	–0.18284000	–0.292095000
H	–0.14829000	–1.22716600	–0.429693000
C	2.90344900	2.72012400	0.074262000
H	3.36934700	3.03715900	1.016942000
H	3.41280700	3.27249700	–0.726467000
H	1.86341200	3.05482300	0.093130000
C	–1.81311100	0.13762500	–0.237813000
H	–2.05020100	1.19724600	–0.102352000
C	–2.88655700	–0.72331400	–0.330381000
C	–4.20757800	–0.19052700	–0.246175000

I

E(scf) = –1707.43842737a.u.

C	13.876730000	0.098320000	–1.02193900
C	12.954400000	0.800519000	–0.01266300
C	11.652642000	–0.004086000	0.06951600
C	11.826628000	–1.447943000	0.20246000
C	13.014566000	–2.036528000	–0.08166800
C	14.192274000	–1.350388000	–0.67622400
H	13.393952000	0.131202000	–2.01084600
H	14.816713000	0.663985000	–1.11743200
C	10.727278000	–2.324502000	0.73534200
H	10.168532000	–1.838533000	1.54332000
H	9.998707000	–2.596855000	–0.03943200
H	11.142972000	–3.262119000	1.12022000
C	12.761357000	2.236998000	–0.49946500
H	12.212921000	2.280003000	–1.44931800
H	12.227943000	2.858930000	0.22974400
H	13.741629000	2.704884000	–0.65609100
C	13.608116000	0.852763000	1.37788600
H	14.578205000	1.365929000	1.32669500
H	12.968223000	1.408792000	2.07537900
H	13.785876000	–0.145255000	1.78743700
H	13.143072000	–3.099595000	0.12708900
H	14.468487000	–1.882031000	–1.60780100
O	15.270736000	–1.486388000	0.25615800
H	16.064607000	–1.148976000	–0.18280900
C	10.446942000	0.663403000	0.09673100
C	9.112682000	0.190038000	0.06330300
H	8.919754000	–0.871825000	–0.06823000
C	7.989114000	1.006013000	0.13637600
C	6.714306000	0.403680000	0.03554700
H	6.715687000	–0.681567000	–0.10634800
C	5.470798000	1.012654000	0.09568700
H	5.418857000	2.092163000	0.24681900
C	4.263349000	0.318931000	–0.02640100
H	4.324414000	–0.761325000	–0.18689600
C	8.137341000	2.490167000	0.31740500
H	8.713201000	2.729203000	1.22002600
H	8.673187000	2.944031000	–0.52663300
H	7.175972000	3.002768000	0.40078300
C	2.980938000	0.877331000	0.04027300
C	1.870370000	0.035422000	–0.11016600
H	2.093734000	–1.021055000	–0.28765000
C	0.519794000	0.377945000	–0.06133200
H	0.241548000	1.417544000	0.12722600
C	–0.519172000	–0.528739000	–0.23697800
H	–0.245469000	–1.567932000	–0.43366700
C	2.831306000	2.353349000	0.27001900
H	3.273017000	2.655252000	1.22853500
H	3.345974000	2.929945000	–0.50935500
H	1.787794000	2.677009000	0.27545700
C	–1.872891000	–0.180228000	–0.17681600
H	–2.086785000	0.872149000	0.03397900
C	–2.980871000	–1.003631000	–0.35084800
C	–4.273757000	–0.436719000	–0.23491900
H	–4.316738000	0.630997000	–0.00010700

H	–4.286796000	0.89265200	–0.11521500
C	–5.36994400	–0.91159000	–0.30912400
H	–5.29698000	–1.99266200	–0.43701600
C	–2.72487900	–2.20219000	–0.51306600
H	–1.67885900	–2.51559500	–0.54456000
H	–3.20915400	–2.75589700	0.30216500
H	–3.19882000	–2.54042900	–1.444201000
C	–6.66319100	–0.33579200	–0.209908000
H	–6.69049000	0.75103100	–0.085244000
C	–7.87615600	–0.97846100	–0.244818000
C	–9.07714000	–0.19782200	–0.109040000
H	–8.9321000	0.87786100	0.011484000
C	–10.34024800	–0.696248000	–0.07250600
H	–10.44189300	–1.784088000	–0.10186800
C	–8.00691400	–2.461618000	–0.40630500
H	–7.04381900	–2.963194000	–0.527637000
H	–8.5049700	–2.912312000	0.463660000
H	–8.62367900	–2.712610000	–1.279352000
C	–11.5876200	0.032806000	0.112352000
C	–12.6148410	–0.702735000	0.984399000
C	–13.1267240	1.984890000	–0.218261000
C	–13.78157500	0.214878000	1.382770000
C	–14.27885200	1.130907000	0.287701000
H	–14.61100800	–0.398914000	1.761002000
H	–13.46241100	0.857018000	2.217991000
C	–13.13816800	–1.942342000	0.240747000
H	–12.33370500	–2.661553000	0.040726000
H	–13.88571300	–2.460595000	0.856470000
H	–13.61065600	–1.670377000	–0.705803000
C	–11.95213700	–1.169959000	2.288181000
H	–11.16582100	–1.912553000	2.106819000
H	–11.49669600	–0.330604000	2.829229000
H	–12.69890800	–1.632262000	2.947868000
C	–11.84474600	1.237297000	–0.462586000
H	–12.94316500	2.801361000	0.502658000
C	–10.90276300	1.944542000	–1.388167000
H	–10.33498800	2.737362000	–0.877076000
H	–10.17259100	1.271211000	–1.847371000
H	–11.46093900	2.445596000	–2.191394000
H	–13.43093200	2.503072000	–1.143403000
H	10.66437900	1.898799000	0.140190000
O	–14.84083000	0.325565000	–0.741496000
H	–15.08150600	0.914644000	–1.469368000
H	–15.05964000	1.794638000	0.704035000

II

E(scf) = –1783.25802236 a.u.

C	13.751843000	–0.253161000	0.47745200
C	12.489284000	0.579604000	0.74091600
C	11.326391000	0.130101000	–0.15073600
C	11.400531000	–0.965515000	–0.95117900
C	12.575921000	–1.901413000	–0.93766600
C	13.495253000	–1.728422000	0.26198600
H	14.239559000	0.109940000	–0.43920300
H	14.471358000	–0.090580000	1.29595900
C	10.373704000	–1.356264000	–1.96853000
H	9.713917000	–2.159580000	–1.60694800
H	9.735564000	–0.520060000	–2.26849000
H	10.881789000	–1.744491000	–2.86021500
C	12.864806000	2.040207000	0.44431300

C	–5.471300000	–1.099691000	–0.38513400
H	–5.445163000	–2.163579000	–0.62613300
C	–2.853960000	–2.465610000	–0.65623300
H	–1.814919000	–2.801319000	–0.69788800
H	–3.366583000	–3.074477000	0.10018200
H	–3.316761000	–2.711244000	–1.62147100
C	–6.729755000	–0.473585000	–0.23953100
H	–6.702303000	0.591257000	0.01158700
C	–7.980904000	–1.037106000	–0.36874800
C	–9.132041000	–0.207473000	–0.15369600
H	–8.921333000	0.829320000	0.11622300
C	–10.428051000	–0.619258000	–0.20684800
H	–10.599897000	–1.682980000	–0.39122300
C	–8.189437000	–2.480128000	–0.70960000
H	–7.251665000	–3.019289000	–0.86557500
H	–8.739360000	–2.999105000	0.08787400
H	–8.790239000	–2.590633000	–1.62204900
C	–11.625121000	0.162259000	0.06512500
C	–12.733829000	–0.622567000	0.78175300
C	–13.000479000	2.261041000	0.04006500
C	–13.823891000	0.311917000	1.32908300
C	–14.226563000	1.438600000	0.40480800
H	–14.707490000	–0.282854000	1.59993700
H	–13.460420000	0.773798000	2.25962600
C	–13.339828000	–1.656138000	–0.18096000
H	–12.589157000	–2.388204000	–0.50517100
H	–14.138850000	–2.216076000	0.32339500
H	–13.768840000	–1.177583000	–1.06414200
C	–12.149703000	–1.374024000	1.98665400
H	–11.424248000	–2.138041000	1.68218100
H	–11.640182000	–0.689931000	2.67735100
H	–12.950832000	–1.880515000	2.54170100
C	–11.776886000	1.460419000	–0.31065200
H	–12.761322000	2.940041000	0.87792700
C	–10.762490000	2.229717000	–1.10078200
H	–10.135792000	2.871821000	–0.46269400
H	–10.086764000	1.581189000	–1.66676800
H	–11.263106000	2.905698000	–1.80776600
H	–13.247664000	2.937830000	–0.79496300
H	10.523023000	1.749667000	0.10670400
O	–14.848825000	0.869512000	–0.74115800
H	–15.051575000	1.594373000	–1.34805600
H	–14.949863000	2.089287000	0.93054700

III_1

E(scf) = –1782.64060457a.u.

C	13.776713000	–0.159677000	0.3163750
C	12.511186000	0.673853000	0.5649990
C	11.312609000	0.119289000	–0.2141070
C	11.370478000	–1.048251000	–0.9089170
C	12.568576000	–1.955302000	–0.8707620
C	13.542090000	–1.652853000	0.2582680
H	14.207843000	0.119640000	–0.6562430
H	14.533195000	0.094936000	1.0757220
C	10.303624000	–1.556905000	–1.8281370
H	9.680092000	–2.329450000	–1.3530420
H	9.633217000	–0.766950000	–2.1790290
H	10.775637000	–2.026416000	–2.7003250
C	12.840560000	2.104232000	0.1081230
H	12.967879000	2.158594000	–0.9802290

H	13.054851000	2.195922000	–0.62502100	H	12.072722000	2.829742000	0.4004600
H	12.091308000	2.748975000	0.76176300	H	13.780028000	2.432118000	0.5726440
H	13.780884000	2.303824000	0.98954100	C	12.160587000	0.718614000	2.0596010
C	12.057765000	0.485245000	2.21217500	H	13.008291000	1.102317000	2.6452250
H	12.880972000	0.781919000	2.87800200	H	11.306968000	1.385213000	2.2374900
H	11.212064000	1.155554000	2.41212600	H	11.892437000	–0.273480000	2.4351200
H	11.744173000	–0.530998000	2.46935400	H	14.498147000	–2.160176000	0.0219210
H	14.453560000	–2.231397000	0.02541400	O	12.983616000	–2.206196000	1.4417700
O	12.868052000	–2.380133000	1.35775100	H	13.53358700	–1.915036000	2.18230900
H	13.374710000	–2.159123000	2.15146400	C	10.113015000	0.940592000	–0.1499340
C	10.135441000	0.965724000	–0.06972300	C	8.824316000	0.503482000	–0.1069310
C	8.846264000	0.537859000	–0.09703500	H	8.637727000	–0.572102000	–0.1014600
H	8.655473000	–0.534501000	–0.17542500	C	7.660110000	1.337711000	–0.0407530
C	7.679357000	1.371737000	–0.00225500	C	6.421845000	0.734901000	0.0199420
C	6.444580000	0.771275000	–0.01344400	H	6.416714000	–0.359397000	0.0282740
H	6.436672000	–0.320675000	–0.08365100	C	5.151781000	1.352315000	0.0595920
C	5.167784000	1.387714000	0.04602300	H	5.100934000	2.442208000	0.0361500
H	5.118098000	2.476491000	0.10103800	C	3.969335000	0.648060000	0.1148490
C	3.993397000	0.683567000	0.03079400	H	4.035353000	–0.443558000	0.1428400
H	4.059313000	–0.407261000	–0.01892700	C	7.841586000	2.823782000	–0.0486110
C	7.866302000	2.853683000	0.09806700	H	8.437450000	3.152141000	0.8143130
H	8.450330000	3.118548000	0.9903800	H	8.386949000	3.151912000	–0.9434180
H	8.426052000	3.241329000	–0.76356900	H	6.894629000	3.368521000	–0.0187150
H	6.920612000	3.398946000	0.15195400	C	2.665896000	1.201879000	0.1304830
C	2.677903000	1.233904000	0.07007600	C	1.574509000	0.340037000	0.1841050
C	1.600778000	0.372993000	0.05506700	H	1.809805000	–0.727942000	0.2244360
H	1.836205000	–0.695001000	0.02031300	C	0.214666000	0.667514000	0.1859300
C	0.225327000	0.694649000	0.07672300	H	–0.079866000	1.718358000	0.1357210
H	–0.070404000	1.746000000	0.10171200	C	–0.807469000	–0.273288000	0.2436210
C	–0.777030000	–0.244338000	0.06416300	H	–0.510466000	–1.323182000	0.3004950
H	–0.477398000	–1.294619000	0.04222800	C	2.510981000	2.691906000	0.0786340
C	2.526180000	2.723519000	0.12557900	H	3.03103600	3.174506000	0.9166090
H	2.998249000	3.135705000	1.02760700	H	2.949395000	3.104371000	–0.8399460
H	3.018059000	3.204016000	–0.73055700	H	1.466828000	3.011671000	0.1155620
H	1.481734000	3.044477000	0.12669500	C	–2.163397000	0.049030000	0.2256320
C	–2.154410000	0.069238000	0.07411000	H	–2.404452000	1.114166000	0.1556340
H	–2.398859000	1.135607000	0.08855800	C	–3.260607000	–0.821780000	0.2799640
C	–3.223100000	–0.802290000	0.06224900	C	–4.550987000	–0.281637000	0.2236520
C	–4.546024000	–0.267737000	0.06015900	H	–4.628251000	0.805502000	0.1321640
H	–4.627998000	0.823121000	0.06928300	C	–5.748609000	–1.001724000	0.2670060
C	–5.707964000	–0.992066000	0.04189000	H	–5.682243000	–2.086234000	0.3686870
H	–5.636165000	–2.080934000	0.03323800	C	–3.088825000	–2.308584000	0.3957770
C	–3.055342000	–2.291303000	0.04579000	H	–2.040429000	–2.615954000	0.3862480
H	–2.008157000	–2.602315000	0.05977300	H	–3.531009000	–2.688130000	1.3262680
H	–3.547333000	–2.754169000	0.91160300	H	–3.590119000	–2.833106000	–0.4275670
H	–3.518196000	–2.731783000	–0.84748900	C	–6.998788000	–0.410371000	0.1793390
C	–6.999302000	–0.403531000	0.02647600	H	–7.011384000	0.678019000	0.0646780
H	–7.020126000	0.690583000	0.02978900	C	–8.267662000	–1.033385000	0.2077190
C	–8.216866000	–1.037556000	0.00031200	C	–9.395823000	–0.233977000	0.0625020
C	–9.411066000	–0.235931000	–0.02397400	H	–9.207575000	0.830316000	–0.0541290
H	–9.255090000	0.844573000	–0.00355800	C	–10.728406000	–0.711262000	0.1012570
C	–10.682870000	–0.713679000	–0.03074200	H	–10.804848000	–1.771226000	0.3402110
H	–10.804102000	–1.797594000	0.03995600	C	–8.401875000	–2.519438000	0.3820160
C	–8.360472000	–2.527748000	–0.00223500	H	–7.436312000	–3.018625000	0.4938930
H	–7.399964000	–3.048864000	0.00630800	H	–8.996273000	–2.764812000	1.2716480
H	–8.928996000	–2.869220000	0.87404700	H	–8.911373000	–2.981114000	–0.4730920
H	–8.917671000	–2.870834000	–0.88408400	C	–11.934163000	–0.063722000	–0.0634920
C	–11.926582000	0.043880000	–0.02091400	C	–13.240881000	–0.833939000	0.1562400
C	–13.041211000	–0.601885000	0.81442800	C	–13.295031000	1.944527000	–0.4021450
C	–13.393892000	1.978205000	–0.65302700	C	–14.200963000	0.067358000	0.9510110

C	–14.213236000	0.367659000	1.04032000
C	–14.601344000	1.192930000	–0.16548000
H	–15.083905000	–0.195924000	1.40339000
H	–13.941702000	1.073723000	1.83971900
C	–13.529280000	–1.886215000	0.12457300
H	–12.726336000	–2.630920000	0.05089900
H	–14.338481000	–2.342575000	0.71043900
H	–13.912666000	–1.681304000	–0.87748600
C	–12.502448000	–0.978683000	2.20199300
H	–11.720922000	–1.746133000	2.14375200
H	–12.074671000	–0.108443000	2.71636600
H	–13.312183000	–1.377579000	2.82797700
C	–12.111353000	1.195410000	–0.71884300
H	–13.250971000	2.857450000	–0.00003800
C	–11.081581000	1.807296000	–1.61918400
H	–10.531759000	2.624920000	–1.12805800
H	–10.337712000	1.083926000	–1.96691600
H	–11.562737000	2.255678000	–2.99607000
H	–13.611334000	2.409052000	–1.64492100
H	10.303891000	2.031719000	0.09679900
O	–15.104230000	0.310384000	–1.16141800
H	–15.295878000	0.841317000	–1.94634300
H	–15.394682000	1.904542000	0.12956900
O	13.301511000	–1.693902000	–2.15286800
H	13.882446000	–2.459020000	–2.27049300
H	12.181255000	–2.934724000	–0.90775400

C	–14.495015000	1.400529000	0.2938820
H	-15.153246000	–0.454709000	1.1148200
H	-13.761594000	0.251395000	1.9421980
C	-13.841270000	–1.203597000	–1.2099510
H	-13.179889000	–1.904541000	–1.7359430
H	-14.820083000	–1.683561000	–1.0825240
H	-13.990669000	–0.324404000	–1.8421270
C	-13.068919000	–2.121914000	0.9611490
H	-12.513102000	–2.890466000	0.4103310
H	-12.553486000	–1.945658000	1.9140590
H	-14.055531000	–2.546271000	1.1859410
C	-12.101039000	1.312157000	–0.5257970
C	-10.989738000	2.051914000	–1.2182620
H	-10.273336000	2.487836000	–0.5101590
H	-10.419637000	1.402941000	–1.8930330
H	-11.395694000	2.883530000	–1.8052070
H	-13.393468000	2.948938000	–0.8247750
H	10.273259000	2.016992000	–0.0626170
O	-15.595039000	1.208825000	–0.6093130
H	-15.654826000	2.001678000	–1.1606460
H	-14.810944000	2.120452000	1.0732090
O	13.227114000	–1.845263000	–2.1356530
H	13.823288000	–2.603776000	–2.2122180
H	12.201170000	–2.989141000	–0.7264710

IV_1

E(scf) = –1858.45883268 a.u.

C	13.981261000	–0.096850000	1.00384000
C	12.706879000	0.758837000	0.94772300
C	11.650215000	0.132602000	0.03000600
C	11.816280000	–1.084226000	–0.55142100
C	12.975427000	–1.983915000	–0.22841000
C	13.731021000	–1.588263000	1.03100300
H	14.587184000	0.095440000	0.10624800
H	14.592739000	0.219639000	1.86392100
C	10.913969000	–1.664485000	–1.59557800
H	10.216595000	–2.406770000	–1.17822900
H	10.315642000	–0.904532000	–2.10702500
H	11.521704000	–2.190097000	–2.34295400
C	13.133257000	2.136900000	0.41720900
H	13.454586000	2.081264000	–0.63015300
H	12.335586000	2.884852000	0.49147900
H	13.978674000	2.513679000	1.00820100
C	12.106102000	0.949178000	2.34856200
H	12.850543000	1.375193000	3.03621500
H	11.253222000	1.639223000	2.31505000
H	11.747517000	0.000243000	2.75856600
H	14.703291000	–2.119106000	1.02104200
O	12.950543000	–2.031190000	2.13240900
H	13.355022000	–1.673430000	2.93499700
C	10.454412000	0.944351000	–0.15172400
C	9.174800000	0.497786000	–0.25089100
H	8.991173000	–0.577704000	–0.21077800
C	8.002893000	1.324276000	–0.34862300
C	6.773260000	0.713998000	–0.38944800
H	6.772791000	–0.379921000	–0.36306400
C	5.493546000	1.324138000	–0.44866500
H	5.437812000	2.413978000	–0.45791000
C	4.322435000	0.614999000	–0.48155200

V_1

E(scf) = –1857.86172744 a.u.

C	13.997507000	0.008034000	0.84497000
C	12.712027000	0.848248000	0.81169200
C	11.630506000	0.188692000	–0.05251600
C	11.792068000	–1.040183000	–0.61149500
C	12.976295000	–1.915273000	–0.31321800
C	13.765882000	–1.484927000	0.91321300
H	14.571898000	0.185794000	–0.07609200
H	14.632659000	0.350611000	1.67747300
C	10.862150000	–1.657423000	–1.60922600
H	10.189715000	–2.397955000	–1.14996800
H	10.236375000	–0.918829000	–2.11905600
H	11.451436000	–2.193985000	–2.36366700
C	13.103622000	2.218005000	0.23504500
H	13.389283000	2.141118000	–0.82127700
H	12.299901000	2.958319000	0.31971000
H	13.964203000	2.618516000	0.78747000
C	12.158341000	1.065337000	2.22813500
H	12.921866000	1.514044000	2.87954800
H	11.297572000	1.745975000	2.20889200
H	11.824168000	0.122679000	2.67209300
H	14.743747000	–2.005095000	0.88476700
O	13.024625000	–1.911934000	2.04727400
H	13.445780000	–1.527183000	2.82845400
C	10.419324000	0.980074000	–0.21025000
C	9.141284000	0.513145000	–0.26052400
H	8.976031000	–0.563665000	–0.18890200
C	7.958452000	1.318598000	–0.34630100
C	6.731930000	0.689154000	–0.33299700
H	6.750337000	–0.403273000	–0.27073700
C	5.448491000	1.277073000	–0.38087400
H	5.375156000	2.364654000	–0.43460700
C	4.279525000	0.548236000	–0.35460900

H	4.391707000	–0.476762000	–0.47586000	H	4.367344000	–0.541070000	–0.30402200
C	8.176831000	2.810881000	–0.38398400	C	8.106779000	2.806219000	–0.43087400
H	8.676889000	3.172725000	0.52525300	H	8.615057000	3.204991000	0.45830000
H	8.810834000	3.114880000	–1.22746900	H	8.721455000	3.094721000	–1.29382200
H	7.228956000	3.347438000	–0.47388900	H	7.148457000	3.323922000	–0.52082900
C	3.005893000	1.163441000	–0.51132700	C	2.965465000	1.074738000	–0.38044400
C	1.930002000	0.301210000	–0.53875800	C	1.889510000	0.192010000	–0.34736800
H	2.166613000	–0.767006000	–0.55040500	H	2.144587000	–0.871594000	–0.31085000
C	0.554422000	0.622776000	–0.54441500	C	0.524314000	0.494239000	–0.35187000
H	0.258627000	1.674027000	–0.51651200	H	0.211431000	1.540596000	–0.38062000
C	–0.448750000	–0.314987000	–0.57509500	C	–0.482550000	–0.464839000	–0.31671700
H	–0.150708000	–1.365432000	–0.60889000	H	–0.168422000	–1.511027000	–0.29107900
C	2.851610000	2.653842000	–0.50200900	C	2.782760000	2.561389000	–0.44105500
H	3.307503000	3.093358000	0.39532200	H	3.275857000	3.057165000	0.40555000
H	3.358059000	3.109456000	–1.36339300	H	3.232187000	2.979072000	–1.35204200
H	1.807123000	2.973335000	–0.53024700	H	1.732177000	2.861336000	–0.42855300
C	–1.825535000	0.000783000	–0.55548400	C	–1.842848000	–0.163535000	–0.30931400
H	–2.067546000	1.066480000	–0.50140200	H	–2.100290000	0.899852000	–0.32935700
C	–2.896289000	–0.867763000	–0.58732500	C	–2.927760000	–1.051432000	–0.27391900
C	–4.217373000	–0.332280000	–0.52770500	C	–4.225129000	–0.527594000	–0.25581200
H	–4.295872000	0.755957000	–0.44907900	H	–4.318091000	0.562193000	–0.27059000
C	–5.381471000	–1.053176000	–0.54685700	C	–5.413373000	–1.264389000	–0.21419400
H	–5.313564000	–2.139088000	–0.62993300	H	–5.333162000	–2.352924000	–0.19966800
C	–2.731845000	–2.354639000	–0.67638400	C	–2.734590000	–2.539850000	–0.24735500
H	–1.685068000	–2.667213000	–0.68874800	H	–1.682903000	–2.832093000	–0.29782800
H	–3.216749000	–2.858438000	0.17018600	H	–3.152053000	–2.978699000	0.66842800
H	–3.203582000	–2.750073000	–1.58602400	H	–3.247451000	–3.022975000	–1.08899600
C	–6.669380000	–0.465660000	–0.44798500	C	–6.670171000	–0.682877000	–0.18298300
H	–6.685107000	0.623710000	–0.34600700	H	–6.696800000	0.411304000	–0.18959400
C	–7.888918000	–1.096510000	–0.44807800	C	–7.931471000	–1.320130000	–0.13568600
C	–9.075711000	–0.299768000	–0.28754100	C	–9.069981000	–0.526259000	–0.08596500
H	–8.911850000	0.773070000	–0.16672400	H	–8.904293000	0.549867000	–0.08409500
C	–10.345343000	–0.776801000	–0.21447100	C	–10.395757000	–1.008010000	0.03345500
H	–10.469331000	–1.862430000	–0.24809300	H	–10.481668000	–2.086595000	0.15964300
C	–8.038776000	–2.580022000	–0.58581200	C	–8.048894000	–2.817587000	–0.12842900
H	–7.083217000	–3.092732000	–0.72105000	H	–7.087278000	–3.315109000	–0.27792600
H	–8.523820000	–3.012852000	0.30037800	H	–8.459926000	–3.183478000	0.82212300
H	–8.675709000	–2.836038000	–1.44283800	H	–8.722884000	–3.170042000	–0.91892300
C	–11.564504000	–0.017788000	0.02743700	C	–11.573207000	–0.292882000	0.08020900
C	–12.579777000	–0.740699000	0.92281000	C	–12.890344000	–0.997581000	0.42411500
C	–13.041069000	1.992234000	–0.17963100	C	–12.786275000	1.809554000	0.18644300
C	–13.682138000	0.207517000	1.42612200	C	–13.585289000	–0.163572000	1.51348500
C	–14.198665000	1.177136000	0.37086700	C	–13.819261000	1.288807000	1.13319700
H	–14.521780000	–0.417597000	1.78341400	H	–14.550608000	–0.612687000	1.78681900
C	–13.218727000	–1.898303000	0.13779900	C	–13.777505000	–1.087721000	–0.82917600
H	–12.457025000	–2.630421000	–0.15754200	H	–13.335293000	–1.764672000	–1.57012200
H	–13.946590000	–2.428249000	0.76698100	H	–14.774179000	–1.477560000	–0.57701400
H	–13.733984000	–1.547707000	–0.75839700	H	–13.892295000	–0.114402000	–1.32322500
C	–11.882298000	–1.338634000	2.15492100	C	–12.714276000	–2.410445000	0.97390300
H	–11.207994000	–2.152641000	1.86359000	H	–12.313838000	–3.100709000	0.22196400
H	–11.303404000	–0.588798000	2.70030800	H	–12.044712000	–2.429654000	1.84244900
H	–12.627751000	–1.755394000	2.84471500	H	–13.686608000	–2.809052000	1.29054700
C	–11.812150000	1.199105000	–0.52258100	C	–11.694936000	1.120240000	–0.24447500
H	–12.771550000	2.760566000	0.56353000	C	–10.731020000	1.827815000	–1.15439100
C	–10.899034000	1.890545000	–1.48740700	H	–9.939723000	2.358165000	–0.60734300
H	–10.291096000	2.667494000	–0.99871200	H	–10.241772000	1.128764000	–1.84028900
H	–10.206902000	1.204002000	–1.98408200	H	–11.260384000	2.585925000	–1.74108800
H	–11.483033000	2.408922000	–2.26104400	H	10.549134000	2.064161000	–0.19543400
H	–13.380783000	2.558357000	–1.06305200	O	–15.127221000	1.569350000	0.59784700
H	10.599155000	2.025882000	–0.11214500	H	–15.232981000	1.017803000	–0.19330400

O	–14.880530000	0.434710000	–0.62519900
H	–15.092202000	1.043744000	–1.34544100
H	–14.912002000	1.866107000	0.86923600
O	13.852696000	–1.980172000	–1.35798000
H	14.434118000	–2.748714000	–1.26880500
H	12.572714000	–3.002310000	–0.06935700
O	–13.145399000	0.976857000	2.49732800
H	–13.867200000	1.516198000	2.84933200

VI

E(scf) = –1857.27296088a.u.

C	14.173573000	–0.039620000	0.55000200
C	12.892842000	0.807157000	0.59799100
C	11.724443000	0.113583000	–0.11453800
C	11.830013000	–1.156325000	–0.63533900
C	12.980186000	–2.015705000	–0.32920100
C	13.949243000	–1.524029000	0.73526700
H	14.660651000	0.097717000	–0.42865600
H	14.887388000	0.347334000	1.29501700
C	10.824273000	–1.789585000	–1.54472600
H	10.105742000	–2.418163000	–0.99988100
H	10.252924000	–1.048546000	–2.11294300
H	11.345830000	–2.462936000	–2.23222100
C	13.226772000	2.137978000	–0.09647300
H	13.388377000	2.000420000	–1.17294300
H	12.449260000	2.898297000	0.03763500
H	14.151386000	2.548686000	0.33046000
C	12.492853000	1.106236000	2.05124200
H	13.317174000	1.606266000	2.57849000
H	11.622588000	1.773860000	2.08849600
H	12.240703000	0.186934000	2.58702600
H	14.890296000	–2.079241000	0.59307800
O	13.361624000	–1.857599000	1.99064300
H	14.000187000	-1.609309000	2.67470300
C	10.524488000	0.915214000	–0.19699900
C	9.232239000	0.481099000	–0.26217300
H	9.032916000	–0.590283000	–0.27648900
C	8.074733000	1.324799000	–0.25071900
C	6.835793000	0.726420000	–0.27698800
H	6.826058000	–0.367040000	–0.31326000
C	5.563015000	1.347862000	–0.26158900
H	5.514251000	2.436773000	–0.21432900
C	4.387125000	0.643996000	–0.30105100
H	4.453463000	–0.446659000	–0.35155200
C	8.262450000	2.809575000	–0.20435800
H	8.810526000	3.110869000	0.69910100
H	8.856006000	3.160687000	–1.05880700
H	7.317055000	3.357607000	–0.21267100
C	3.073664000	1.195767000	–0.28091800
C	1.997227000	0.333851000	–0.33304000
H	2.234337000	–0.732592000	–0.39276800
C	0.622317000	0.653797000	–0.31679300
H	0.323746000	1.702352000	–0.24930700
C	–0.376307000	–0.288131000	–0.37996100
H	–0.071241000	–1.334667000	–0.45102100
C	2.920372000	2.684016000	–0.20015900
H	3.376308000	3.079602000	0.71729300
H	3.425641000	3.180499000	–1.03908200
H	1.875876000	3.004372000	–0.21214100
C	–1.754602000	0.016963000	–0.35247400
H	–2.006278000	1.078169000	–0.26690400

H	–13.806253000	1.910791000	2.04066100
O	13.815135000	–1.914198000	–1.47187500
H	14.416416000	–2.667997000	–1.38812800
H	12.597308000	–2.937757000	–0.12518000
H	–12.958904000	–0.195635000	2.41632900
O	–13.005045000	3.091781000	–0.21162800
H	–13.938290000	3.274806000	0.00511100

VII

E(scf) = –1856.67665269 a.u.

C	14.170726000	0.078747000	0.48800700
C	12.877594000	0.907082000	0.52890500
C	11.710438000	0.174818000	–0.14733800
C	11.832414000	–1.108948000	–0.63423100
C	12.997437000	–1.941036000	–0.31604400
C	13.971668000	–1.402808000	0.72093800
H	14.642505000	0.193750000	–0.50092000
H	14.888358000	0.498962000	1.21117700
C	10.829674000	–1.781458000	–1.51839700
H	10.126849000	–2.408956000	–0.95222500
H	10.240759000	–1.064403000	–2.09921000
H	11.357174000	–2.462348000	–2.19393800
C	13.180417000	2.222023000	–0.20809400
H	13.331937000	2.055443000	–1.28191300
H	12.391438000	2.972662000	–0.08697900
H	14.102627000	2.660504000	0.19574000
C	12.493392000	1.241159000	1.97873400
H	13.315662000	1.771711000	2.47876000
H	11.611088000	1.892940000	2.00984600
H	12.266844000	0.333011000	2.54433000
H	14.919786000	–1.947448000	0.58398000
O	13.405383000	–1.706756000	1.99364900
H	14.042847000	–1.415066000	2.66135000
C	10.497771000	0.951359000	–0.23886800
C	9.209938000	0.491058000	–0.28435900
H	9.032451000	–0.584059000	–0.26410500
C	8.040632000	1.309926000	–0.29437500
C	6.807097000	0.685966000	–0.28448700
H	6.820621000	–0.408144000	–0.27505700
C	5.529936000	1.281169000	–0.28295800
H	5.459541000	2.370131000	–0.28837100
C	4.357797000	0.552769000	–0.27226700
H	4.444739000	–0.537661000	–0.26793400
C	8.196334000	2.799271000	–0.30733800
H	8.731601000	3.148992000	0.58637500
H	8.787593000	3.128750000	–1.17182800
H	7.239276000	3.325718000	–0.34281000
C	3.047188000	1.081832000	–0.26429900
C	1.970266000	0.197167000	–0.25405200
H	2.225739000	–0.866800000	–0.25576100
C	0.606821000	0.499658000	–0.24080600
H	0.293317000	1.546076000	–0.23573700
C	–0.399593000	–0.461976000	–0.23237900
H	–0.084404000	–1.507973000	–0.24010200
C	2.864650000	2.569680000	–0.26622400
H	3.341519000	3.029315000	0.60964400
H	3.330038000	3.025223000	–1.15038200
H	1.813916000	2.868994000	–0.26033400
C	–1.758843000	–0.161775000	–0.21360500
H	–2.017544000	0.901341000	–0.20331500

C	–2.816481000	–0.861640000	–0.41495000
C	–4.142939000	–0.340595000	–0.35027800
H	–4.233868000	0.744484000	–0.24768600
C	–5.297989000	–1.074910000	–0.39479400
H	–5.216094000	–2.157873000	–0.49946100
C	–2.636158000	–2.343581000	–0.54487100
H	–1.586661000	–2.646219000	–0.55684900
H	–3.122690000	–2.875258000	0.28325100
H	–3.096513000	–2.717205000	–1.46919100
C	–6.594450000	–0.506540000	–0.29947400
H	–6.628447000	0.580897000	–0.18447600
C	–7.803124000	–1.157878000	–0.32157700
C	–9.005255000	–0.380700000	–0.17541600
H	–8.862104000	0.694461000	–0.05294600
C	–10.265414000	–0.883578000	–0.12175800
H	–10.367256000	–1.971235000	–0.15377900
C	–7.927074000	–2.641911000	–0.47642600
H	–6.962476000	–3.138626000	–0.60534800
H	–8.415319000	–3.092212000	0.39912900
H	–8.549744000	–2.898375000	–1.34355400
C	–11.508042000	–0.153620000	0.09457800
C	–12.509206000	–0.888258000	0.99465900
C	–13.043714000	1.804516000	–0.19449200
C	–13.652605000	0.037934000	1.43171000
C	–14.180184000	0.940389000	0.34474000
C	–13.058346000	–2.127154000	0.26922800
H	–12.263258000	–2.851697000	0.05130200
H	–13.791333000	–2.638503000	0.90742800
H	–13.554515000	–1.857239000	–0.66555200
C	–11.803016000	–1.355361000	2.27599400
H	–11.024224000	–2.099125000	2.06847300
H	–11.330588000	–0.515577000	2.80079500
H	–12.528664000	–1.816987000	2.95890700
C	–11.769513000	1.054374000	–0.46875200
C	–10.838205000	1.788027000	–1.38313100
H	–10.252006000	2.544452000	–0.84218500
H	–10.132036000	1.124940000	–1.89267200
H	–11.406434000	2.337228000	–2.14655200
H	–13.384570000	2.256282000	–1.15122400
H	10.668303000	1.991368000	–0.09371700
O	–14.761878000	0.147471000	–0.67763300
H	–15.005048000	0.739260000	–1.40360200
H	–14.943526000	1.618341000	0.77920900
O	13.148233000	–3.107946000	–0.86251900
H	–13.289391000	0.701028000	2.23002100
H	–14.468214000	–0.562930000	1.85703400
O	–12.708430000	2.847021000	0.72336700
H	–13.480870000	3.427150000	0.79409400

3

E(scf) = –1856.09491320 a.u.

C	14.123510000	–0.154434000	0.42213700
C	12.854326000	0.665913000	0.69462300
C	11.672710000	0.179670000	–0.15212700
C	11.742616000	–0.942142000	–0.94483900
C	12.884337000	–1.863374000	–0.86854000
C	13.888000000	–1.639808000	0.25301100
H	14.593679000	0.210184000	–0.5048880
H	14.855110000	0.038595000	1.22333400
C	10.715295000	–1.337316000	–1.95924200
H	10.014556000	–2.089334000	–1.57058900

C	–2.843053000	–1.052436000	–0.20493700
C	–4.139791000	–0.529272000	–0.17988000
H	–4.233196000	0.560422000	–0.16618300
C	–5.328287000	–1.267440000	–0.16821100
H	–5.248447000	–2.355888000	–0.18185400
C	–2.648465000	–2.540585000	–0.22426000
H	–1.595352000	–2.831332000	–0.21422400
H	–3.125964000	–3.016044000	0.64211100
H	–3.101556000	–2.988726000	–1.11823600
C	–6.584333000	–0.685576000	–0.13627700
H	–6.610223000	0.408467000	–0.12024900
C	–7.846502000	–1.322905000	–0.11673200
C	–8.983986000	–0.527723000	–0.07092300
H	–8.817313000	0.548151000	–0.05128900
C	–10.311938000	–1.008696000	0.02303300
H	–10.402374000	–2.087960000	0.13925600
C	–7.966600000	–2.819845000	–0.13655600
H	–7.002453000	–3.317525000	–0.26760100
H	–8.403546000	–3.199352000	0.79684700
H	–8.619973000	–3.157909000	–0.95026700
C	–11.487190000	–0.289145000	0.05708000
C	–12.811389000	–0.990062000	0.38089900
C	–12.693158000	1.816921000	0.15149600
C	–13.516796000	–0.156374000	1.46371700
C	–13.739488000	1.298360000	1.08521600
C	–13.682250000	–1.072372000	–0.88419100
H	–13.232023000	–1.746361000	–1.62297900
H	–14.682244000	–1.462183000	–0.64606700
H	–13.789927000	–0.096558000	–1.37489000
C	–12.647453000	–2.405317000	0.92828200
H	–12.240732000	–3.094804000	0.17903200
H	–11.988980000	–2.430155000	1.80509500
H	–13.625061000	–2.801152000	1.23157800
C	–11.598059000	1.124638000	–0.26606400
C	–10.619876000	1.829728000	–1.16271500
H	–9.839260000	2.364664000	–0.60493900
H	–10.117987000	1.128220000	–1.83680300
H	–11.140147000	2.583792000	–1.76258100
H	10.621380000	2.032373000	–0.16221700
O	–15.039102000	1.588135000	0.53660200
H	–15.142178000	1.037148000	–0.25531100
H	–13.732076000	1.917537000	1.99458800
O	13.176771000	–3.046744000	–0.81831600
H	–12.902873000	–0.194221000	2.37477700
H	–14.487482000	–0.602152000	1.72274000
O	–12.901983000	3.098743000	–0.24871700
H	–13.835681000	3.288358000	–0.03897700

III

E(scf) = –1782.65811824 a.u.

C	13.482304000	0.279479000	–1.01878800
C	12.658004000	0.890954000	0.12464000
C	11.304790000	0.170566000	0.14521500
C	11.376745000	–1.281159000	0.17511500
C	12.515801000	–1.899770000	–0.23721600
C	13.655988000	–1.227927000	–0.92508400
H	12.983438000	0.517010000	–1.96992600
H	14.474832000	0.753474000	–1.06124200
C	10.296122000	–2.134068000	0.77569400
H	9.732752000	–1.586123000	1.53779800

H	10.126603000	–0.484067000	–2.3093100	H	9.574282000	–2.494114000	0.02985300
H	11.220430000	–1.813776000	–2.80546400	H	10.736368000	–3.025071000	1.23497700
C	13.198264000	2.124710000	0.35053300	C	12.554407000	2.391071000	–0.13970800
H	13.341790000	2.262591000	–0.72851100	H	11.988281000	2.610161000	–1.05377200
H	12.435190000	2.834897000	0.68840100	H	12.076787000	2.924423000	0.69077500
H	14.135230000	2.403141000	0.85037500	H	13.559754000	2.815846000	–0.25592200
C	12.465042000	0.599873000	2.17979900	C	13.381574000	0.690063000	1.46553800
H	13.294855000	0.953857000	2.80708300	H	14.367534000	1.174042000	1.44474900
H	11.598045000	1.240953000	2.38410500	H	12.803385000	1.136979000	2.28378700
H	12.213193000	–0.422000000	2.47656700	H	13.537356000	–0.368623000	1.69383100
H	14.819262000	–2.148437000	–0.04343000	H	13.728137000	–1.648380000	–1.94654700
O	13.329844000	–2.263136000	1.40728500	O	14.825493000	–1.649949000	–0.19773200
H	13.980856000	–2.174340000	2.11818700	H	15.598068000	–1.420096000	–0.73247200
C	10.486353000	1.004896000	–0.06275500	C	10.137009000	0.900792000	0.21302100
C	9.193334000	0.583451000	–0.15295200	C	8.795987000	0.464337000	0.09669000
H	8.998305000	–0.479323000	–0.30100100	H	8.600773000	–0.582547000	–0.12942300
C	8.031905000	1.413586000	–0.02430500	C	7.679811000	1.286290000	0.19793600
C	6.797480000	0.810152000	–0.08779700	C	6.401106000	0.707934000	0.03222900
H	6.795055000	–0.275983000	–0.21865700	H	6.394314000	–0.366677000	–0.17579400
C	5.519919000	1.418040000	–0.01257200	C	5.162043000	1.323726000	0.11105700
H	5.462273000	2.502170000	0.09663800	H	5.116732000	2.394045000	0.31987400
C	4.351468000	0.704451000	–0.07641300	C	3.951871000	0.643117000	–0.05385700
H	4.429110000	–0.381829000	–0.17679600	H	4.011172000	–0.430792000	–0.25378400
C	8.213021000	2.887234000	0.16655000	C	7.841237000	2.751275000	0.48911300
H	8.775805000	3.100639000	1.08572300	H	8.296199000	2.918199000	1.47408700
H	8.788157000	3.326915000	–0.65882500	H	8.498247000	3.234202000	–0.24574100
H	7.264429000	3.425934000	0.23256700	H	6.891057000	3.291174000	0.47446700
C	3.031863000	1.240134000	–0.02569100	C	2.669890000	1.200306000	0.02032300
C	1.966368000	0.365089000	–0.08452500	C	1.560642000	0.360468000	–0.15142800
H	2.216161000	–0.697285000	–0.15990100	H	1.787765000	–0.693675000	–0.33841500
C	0.588121000	0.668472000	–0.05804300	C	0.208257000	0.695621000	–0.10719700
H	0.277376000	1.713831000	0.00359000	H	–0.078340000	1.734273000	0.07468300
C	–0.399214000	–0.286421000	–0.10676500	C	–0.820276000	–0.225118000	–0.27386700
H	–0.082868000	–1.330249000	–0.16354100	H	–0.529262000	–1.263354000	–0.44995100
C	2.861477000	2.723851000	0.09094400	C	2.517540000	2.669503000	0.28803500
H	3.309622000	3.101219000	1.01974800	H	2.940454000	2.943245000	1.26359600
H	3.364646000	3.245511000	–0.73350600	H	3.048284000	3.267684000	–0.46385200
H	1.813754000	3.033378000	0.08391500	H	1.473914000	2.993540000	0.28337900
C	–1.779868000	0.005576000	–0.08573500	C	–2.180239000	0.098849000	–0.22400500
H	–2.042202000	1.066407000	–0.03667800	H	–2.417014000	1.153432000	–0.05176300
C	–2.833338000	–0.885565000	–0.11997900	C	–3.269128000	–0.757351100	–0.35879200
C	–4.162318000	–0.371530000	–0.09349400	C	–4.577503000	–0.221531000	–0.27058400
H	–4.261928000	0.716837000	–0.05132400	H	–4.65345700	0.857936000	–0.10907100
C	–5.313638000	–1.115446000	–0.11417400	C	–5.751630000	–0.935419000	–0.36257700
H	–5.226485000	–2.202145000	–0.15365500	H	–5.683403000	–2.013441000	–0.51717400
C	–2.640741000	–2.370059000	–0.18418300	C	–3.106162000	–2.230124000	–0.58802000
H	–1.588982000	–2.664677000	–0.17682300	H	–2.059421000	–2.541375000	–0.62100200
H	–3.126565000	–2.868640000	0.66476800	H	–3.595365000	–2.810651000	0.20542100
H	–3.093363000	–2.788163000	–1.09307000	H	–3.571460000	–2.539517000	–1.53341800
C	–6.6099070000	–0.545273000	–0.08379500	C	–7.035569000	–0.354215000	–0.25922000
H	–6.647325000	0.547639000	–0.05209600	H	–7.056908000	0.730271000	–0.11296100
C	–7.820206000	–1.199765000	–0.08780500	C	–8.258429000	–0.987512000	–0.31286800
C	–9.017214000	–0.412505000	–0.05266500	C	–9.450956000	–0.201357000	–0.17018500
H	–8.872879000	0.668244000	–0.02724300	H	–9.299208000	0.871974000	–0.03656400
C	–10.285011000	–0.912138000	0.01580100	C	–10.720081000	–0.691283000	–0.13961600
H	–10.383826000	–1.996295000	0.08549300	H	–10.828952000	–1.778194000	–0.17742000
C	–7.9416440000	–2.691694000	–0.12266800	C	–8.397175000	–2.467169000	–0.49916300
H	–6.9743540000	–3.195814000	–0.18554500	H	–7.436330000	–2.971341000	–0.62797100
H	–8.451004000	–3.068004000	0.77520500	H	–8.897378000	–2.930150000	0.36324100
H	–8.542110000	–3.019633000	–0.98112800	H	–9.014737000	–2.701362000	–1.37634300

C	–11.523398000	–0.177136000	0.09191300
C	–12.698584000	–0.890599000	0.77954500
C	–13.070587000	1.616281000	–0.51416700
C	–13.550855000	0.155706000	1.50643600
C	–14.023232000	1.293679000	0.62771000
C	–13.529481000	–1.621492000	–0.28394500
H	–12.914794000	–2.346050000	–0.83485700
H	–14.355353000	–2.168896000	0.18880100
H	–13.968278000	–0.924853000	–1.00612800
C	–12.237674000	–1.905196000	1.83082000
H	–11.782007000	–2.799599000	1.38994100
H	–11.514941000	–1.468580000	2.53163700
H	–13.103344000	–2.245902000	2.41262700
C	–11.728412000	1.033459000	–0.53250600
C	–10.748363000	1.718025000	–1.43139600
H	–10.097464000	2.415572000	–0.88597500
H	–10.094425000	1.007048000	–1.94926800
H	–11.299323000	2.307311000	–2.17096600
H	10.647301000	2.054917000	0.18672900
O	–15.301866000	1.009662000	0.09346400
H	–15.383636000	1.615923000	–0.66504700
H	–14.0718300	2.220253000	1.23442300
O	13.018693000	–2.806585000	–1.64076900
H	–12.954819000	0.565135000	2.33422200
H	–14.429081000	–0.324487000	1.95841100
O	–13.488916000	2.367204000	–1.39857200

IV

E(scf) = –1782.07233750 a.u.

C	–13.867200000	0.016382000	0.52087300
C	–12.581750000	–0.823850000	0.56031900
C	–11.413644000	–0.111077000	–0.13396400
C	–11.525748000	1.165540000	–0.63682700
C	–12.682482000	2.012622000	–0.32253700
C	–13.651145000	1.498718000	0.73182900
H	–14.349605000	–0.107228000	–0.4618740
H	–14.581837000	–0.386933000	1.25639600
C	–10.520500000	1.818727000	–1.53240500
H	–9.808645000	2.444174000	–0.97531700
H	–9.941729000	1.089806000	–2.10871600
H	–11.043887000	2.498355000	–2.21231700
C	–12.903494000	–2.143619000	–0.16029800
H	–13.061807000	–1.986838000	–1.23461900
H	–12.121173000	–2.900901000	–0.03740600
H	–13.826889000	–2.568534000	0.25515600
C	–12.188465000	–1.146540000	2.01032300
H	–13.011975000	–1.662892000	2.52287600
H	–11.313020000	–1.807607000	2.04118600
H	–11.947329000	–0.235038000	2.56426200
H	–14.594805000	2.051440000	0.59754600
O	–13.067290000	1.813698000	1.99377500
H	–13.703802000	1.546337000	2.67253800
C	–10.207666000	–0.902820000	–0.22121900
C	–8.917943000	–0.459325000	–0.27671300
H	–8.725550000	0.613383000	–0.27957400
C	–7.755237000	–1.295614000	–0.26688000
C	–6.519602000	–0.689690000	–0.28124200
H	–6.515886000	0.403974000	–0.31209800
C	–5.244150000	–1.304893000	–0.25671400

C	–11.960955000	0.045180000	0.05229900
C	–12.995608000	–0.691931000	0.91433500
C	–13.483774000	2.014842000	–0.25484800
C	–14.153803000	0.231193000	1.32472600
C	–14.643437000	1.165411000	0.24164400
H	–14.988610000	–0.379657000	1.69588900
H	–13.828347000	0.860013000	2.16765700
C	–13.529999000	1.916386000	0.15412200
H	–12.731114000	–2.638369000	–0.05794500
H	–14.280582000	–2.437906000	0.76343100
H	–14.001776000	–1.626160000	–0.78731500
C	–12.338455000	–1.182018000	2.21263200
H	–11.559748000	–1.930302000	2.02257500
H	–11.874654000	–0.353966000	2.76390500
H	–13.090565000	–1.644427000	2.86624100
C	–12.208360000	1.259164000	–0.50820700
H	–13.293466000	2.821597000	0.47532200
C	–11.260616000	1.968735000	–1.42605000
H	–10.686841000	2.752275000	–0.90728200
H	–10.535566000	1.294490000	–1.89194400
H	–11.814731000	2.481949000	–2.22452200
H	–13.783752000	2.546270000	–1.17397300
H	10.255096000	1.976844000	0.33105400
O	–15.214440000	0.378122000	–0.79678300
H	–15.447317000	0.978152000	–1.51822600
H	–15.417649000	1.831260000	0.66688600
O	12.675460000	–3.242690000	–0.09161700
H	13.635041000	–3.399591000	–0.15519900

V

E(scf) = –1781.45521977 a.u.

C	–13.867603000	0.013867000	0.46475400
C	–12.585190000	–0.831427000	0.49388300
C	–11.408891000	–0.104801000	–0.17243000
C	–11.513368000	1.186496000	–0.64150900
C	–12.669867000	2.028384000	–0.31414100
C	–13.648970000	1.489452000	0.71776200
H	–14.340923000	–0.081665000	–0.52544900
H	–14.590511000	–0.406757000	1.18229300
C	–10.500750000	1.859119000	–1.51400300
H	–9.792073000	2.469943000	–0.93702200
H	–9.919059000	1.143396000	–2.10356700
H	–11.018336000	2.555757000	–2.18104000
C	–12.904674000	–2.132265000	–0.26090100
H	–13.054781000	–1.949323000	–1.33226000
H	–12.125054000	–2.894224000	–0.15047900
H	–13.832005000	–2.564623000	0.13760700
C	–12.204941000	–1.189750000	1.93901000
H	–13.033313000	–1.717490000	2.43177300
H	–11.330414000	–1.852320000	1.96119600
H	–11.968093000	–0.291986000	2.51680000
H	–14.589991000	2.048369000	0.59087100
O	–13.074481000	1.768760000	1.99237100
H	–13.713472000	1.477379000	2.65876900
C	–10.206014000	–0.896111000	–0.27496000
C	–8.912248000	–0.453362000	–0.31089300
H	–8.718364000	0.618444000	–0.27388500
C	–7.755234000	–1.290544000	–0.32988400
C	–6.512395000	–0.686800000	–0.30383800
H	–6.507342000	0.407024000	–0.27704600
C	–5.245335000	–1.304471000	–0.30348200

H	–5.191704000	–2.393502000	–0.20722000	H	–5.194476000	–2.394163000	–0.32605600
C	–4.069791000	–0.597686000	–0.28719100	C	–4.060871000	–0.598182000	–0.27194900
H	–4.137750000	0.492631000	–0.34241200	H	–4.127352000	0.493418000	–0.25089200
C	–7.934182000	–2.781885000	–0.23376100	C	–7.934684000	–2.776800000	–0.36692900
H	–8.482435000	–3.094589000	0.66570300	H	–8.480509000	–3.131151000	0.51846400
H	–8.523914000	–3.128582000	–1.09264500	H	–8.526260000	–3.083279000	–1.23953300
H	–6.985644000	–3.324390000	–0.24559600	H	–6.986219000	–3.318167000	–0.40530500
C	–2.756145000	–1.147709000	–0.24945800	C	–2.759915000	–1.152492000	–0.26129100
C	–1.678409000	–0.286786000	–0.29774400	C	–1.667489000	–0.288502000	–0.22912200
H	–1.913126000	0.779062000	–0.37514200	H	–1.902565000	0.780079000	–0.21835300
C	–0.304993000	–0.610142000	–0.25665500	C	–0.309698000	–0.617707000	–0.20810700
H	–0.012348000	–1.658150000	–0.15999900	H	–0.016548000	–1.669925000	–0.21288000
C	0.701538000	0.322936000	–0.32964600	C	0.714371000	0.323674000	–0.18017500
H	0.408100000	1.370035000	–0.43494800	H	0.420054000	1.375651000	–0.17951000
C	–2.603780000	–2.635456000	–0.15531100	C	–2.604838000	–2.643202000	–0.28619600
H	–3.066968000	–3.024209000	0.76141000	H	–3.107066000	–3.109559000	0.57156500
H	–3.102614000	–3.137961000	–0.99451500	H	–3.060843000	–3.073751000	–1.18760500
H	–1.559494000	–2.956494000	–0.15770600	H	–1.559999000	–2.961767000	–0.26554000
C	2.075554000	0.003355000	–0.27339500	C	2.067771000	–0.004165000	–0.15376000
H	2.311563000	–1.057281000	–0.14531300	H	2.304953000	–1.072325000	–0.15067000
C	3.151262000	0.863375000	–0.35757500	C	3.168606000	0.864566000	–0.13105200
C	4.467435000	0.323702000	–0.25783300	C	4.454867000	0.314355000	–0.10116800
H	4.536759000	–0.756354000	–0.09893000	H	4.526057000	–0.776995000	–0.09167900
C	5.638785000	1.029054000	–0.33794700	C	5.656920000	1.028839000	–0.08297200
H	5.583383000	2.105781000	–0.50689500	H	5.597803000	2.118346000	–0.09794300
C	2.995107000	2.341172000	–0.54961200	C	3.003804000	2.356318000	–0.14032300
H	1.950164000	2.658786000	–0.57674600	H	1.956524000	2.667314000	–0.14354700
H	3.488808000	2.898539000	0.25707100	H	3.477307000	2.814441000	0.73747400
H	3.462685000	2.669232000	–1.48753400	H	3.478944000	2.803248000	–1.02325400
C	6.918957000	0.432970000	–0.20522500	C	6.901887000	0.422693000	–0.04778400
H	6.919882000	–0.643959000	–0.01137300	H	6.906192000	–0.671602000	–0.03035000
C	8.148648000	1.040197000	–0.28323000	C	8.175646000	1.036535000	–0.03219400
C	9.323504000	0.235278000	–0.08352600	C	9.298013000	0.216932000	0.00678900
H	9.138359000	–0.815185000	0.14876600	H	9.100934000	–0.851344000	0.03402000
C	10.605688000	0.684769000	–0.10750900	C	10.631096000	0.692159000	0.05466000
H	10.749799000	1.757606000	–0.25994100	H	10.701542000	1.777342000	0.10378800
C	8.319025000	2.502551000	–0.55498500	C	8.321993000	2.530993000	–0.05950000
H	7.367717000	3.022811000	–0.69119800	H	7.360057000	3.048254000	–0.09348600
H	8.851989000	2.998189000	0.26817400	H	8.856262000	2.896150000	0.82705800
H	8.919864000	2.671011000	–1.45846200	H	8.899769000	2.860192000	–0.93217700
C	11.821907000	–0.071156000	0.15431800	C	11.840433000	0.031525000	0.09467200
C	12.907142000	0.726833000	0.89152600	C	13.133662000	0.842006000	0.23344800
C	13.249987000	–2.133259000	0.09386700	C	13.210208000	–1.991401000	0.25739700
C	14.014159000	–0.192122000	1.43138800	C	14.046440000	0.129626000	1.24592300
C	14.452609000	–1.290721000	0.48990000	C	14.371986000	–1.307008000	0.89249300
C	13.493372000	1.788365000	–0.05272200	C	13.812016000	0.935909000	–1.14343400
H	12.728227000	2.511391000	–0.36348000	H	13.182083000	1.508403000	–1.83677100
H	14.282234000	2.354667000	0.46027700	H	14.781014000	1.444816000	–1.05965500
H	13.929811000	1.333516000	–0.94465000	H	13.999274000	–0.050635000	–1.57599300
C	12.299852000	1.446234000	2.10459400	C	12.918993000	2.264622000	0.75130300
H	11.561241000	2.200831000	1.80809900	H	12.396685000	2.901284000	0.02677200
H	11.800566000	0.740873000	2.78124000	H	12.351250000	2.280003000	1.69067800
H	13.087006000	1.960065000	2.67255200	H	13.892452000	2.733952000	0.94040200
C	12.009267000	–1.355947000	–0.24933100	C	12.028994000	–1.408011000	–0.06573200
C	11.020566000	–2.134716000	–1.06239800	C	10.955443000	–2.286206000	–0.64565400
H	10.40018900	–2.798491000	–0.44061900	H	10.191304000	–2.553016000	0.09575200
H	10.337864000	–1.492729000	–1.62743600	H	10.436820000	–1.806919000	–1.48391000
H	11.543380000	–2.790052000	–1.77247300	H	11.386965000	–3.227164000	–1.00464400
H	13.520924000	–2.784327000	–0.75396600	H	–10.344266000	–1.976434000	–0.21617200
H	–10.344198000	–1.981015000	–0.1303200	O	15.519179000	–1.296233000	0.02955100

O	15.074889000	–0.684703000	–0.63670200
H	15.314555000	–1.393384000	–1.24929200
H	15.184150000	–1.935345000	1.01141600
O	–12.856508000	3.111239000	–0.84070000
H	13.652609000	–0.680002000	2.34925300
H	14.880684000	0.418037000	1.72210100
H	13.018661000	–2.837029000	0.91321500
H	15.620034000	–2.190433000	–0.32615200
H	14.640999000	–1.850296000	1.81856400
O	–12.836657000	3.140725000	–0.80502900
H	13.552619000	0.148041000	2.22844300
H	14.989496000	0.682499000	1.35124200
H	13.327081000	–3.061615000	0.06188000
